# Structure-Based Druggability Assessment of the Mammalian Structural Proteome with Inclusion of Light Protein Flexibility

**DOI:** 10.1371/journal.pcbi.1003741

**Published:** 2014-07-31

**Authors:** Kathryn A. Loving, Andy Lin, Alan C. Cheng

**Affiliations:** 1 Schrödinger LLC, New York, New York, United States of America; 2 Amgen Inc., South San Francisco, California, United States of America; Icahn School of Medicine at Mount Sinai, United States of America

## Abstract

Advances reported over the last few years and the increasing availability of protein crystal structure data have greatly improved structure-based druggability approaches. However, in practice, nearly all druggability estimation methods are applied to protein crystal structures as rigid proteins, with protein flexibility often not directly addressed. The inclusion of protein flexibility is important in correctly identifying the druggability of pockets that would be missed by methods based solely on the rigid crystal structure. These include cryptic pockets and flexible pockets often found at protein-protein interaction interfaces. Here, we apply an approach that uses protein modeling in concert with druggability estimation to account for light protein backbone movement and protein side-chain flexibility in protein binding sites. We assess the advantages and limitations of this approach on widely-used protein druggability sets. Applying the approach to all mammalian protein crystal structures in the PDB results in identification of 69 proteins with potential druggable cryptic pockets.

## Author Summary

Advances reported over the last few years and the increasing availability of protein crystal structure data have greatly improved structure-based druggability approaches. These algorithms predict our ability to discover small molecule drugs for protein targets and can help in identifying promising new biological targets for small molecule drug discovery. However, in practice, nearly all druggability estimation methods are applied to protein crystal structures as rigid proteins, with protein flexibility often not directly addressed. The increasing interest in finding small molecule drugs to protein-protein interfaces makes this issue particularly acute since these interfaces tend to have substantial flexibility compared to traditional enzyme targets. Here, we apply an approach that accounts for light protein backbone movement and protein side-chain flexibility in protein binding sites. We present the results of applying this method to all publicly available mammalian protein crystal structures.

## Introduction

The majority of small molecule drug discovery efforts towards new, unprecedented biological targets do not progress past high-throughput screening or hit-to-lead optimization due to lack of pursuable chemical matter [1], [2]. To counter this, drug discovery groups increasingly use druggability analysis methods to estimate the amenability of new targets to small molecule drug discovery efforts. In prioritizing new targets, druggability analysis results are then considered along with the strength of evidence that affecting the target will lead to human therapeutic benefit [3]. The results also inform the use of structure-based drug design resources and alternative approaches, such as those involving pro-drugs and covalent interactions, for targets that are expected to be very difficult.

In a drug discovery setting, small molecule druggability is commonly defined as whether a small molecule can bind a desired biological site with good, nanomolar range potency, and, at the same time, also have good, drug-like properties conducive to oral bioavailability and clinical progression [3]–[6]. Thus, the concept refers to chemical tractability of the target. The term, bindability, is also used [7], although the term may not capture the desire for the optimized compound to have drug-like properties. We emphasize that a binding site is not necessarily druggable simply because a ligand binds; the ligand additionally needs to have reasonable drug-like properties and potency. The concepts of ‘druglikeness’ and ‘druggability’ as we use it here cover the most common strategies for small molecule drug discovery, and alternative strategies (e.g., involving covalent adducts, metal chelation, prodrugs, and non-oral delivery) can also be useful in prosecuting targets that are found to be likely ‘undruggable’ when only weak, non-covalent interactions are considered [4].

Druggability estimation has historically been based on precedence, that is, whether there are known drugs targeting the protein or one of its homologs [2], [3]. However, this type of data is scarce or non-existent for many newer protein targets. Advances reported over the last few years allow us to leverage the increasing availability of protein crystal structure data using structure-based druggability approaches. There are at least ten published methods for estimating druggability this way [3], [6], and the body of work is extremely consistent in finding that druggable sites are those that have particular ranges of size, curvature, and hydrophobic character [3]–[6]. These descriptors largely characterize aspects of receptor desolvation [4], and atomistic simulations using molecular dynamics have now shown desolvation to be relevant and sufficient for predicting druggability [8], [9].

Many current structure-based methods for druggability estimation are remarkably accurate if the potential small molecule binding site is largely rigid [3], [6]. Binding sites are not always rigid though, and druggability methods are less accurate if the protein readily changes conformation upon small molecule binding. This is particularly true of protein-protein interface and allosteric sites, where druggable pockets often become exposed only with protein movement [10]–[12]. These ‘cryptic pockets’ are large and shallow when bound to their biological peptide or protein partners, but tend to have high hydrophobic character, and, crucially, have flexibility such that larger, deeper pockets more typical of druggable binding sites are energetically accessible [10], [11].

To begin to address these sites, we apply an approach to modeling conservative movements in pockets using comparative protein modeling approaches coupled closely with structure-based druggability analysis. The approach models relatively light protein motions, involving side-chain flexibility and local protein backbone movements, and maintains reasonable prediction accuracy in retrospective validation studies. It allows us to take pockets that a rigid-protein druggability analysis would deem to have some drug-like properties, but not have sufficient drug-like size, and assess whether local protein motion can result in the pocket having all the drug-like properties, including drug-like size. The approach is computationally efficient enough to enable mining of the structural proteome while taking into account light protein flexibility. Applying the method to roughly 18,000 mammalian protein crystal structures in the PDB results in prediction of one percent of proteins as containing likely druggable cryptic pockets.

## Results and Discussion

We combine a druggability scoring model with protein modeling and docking methods to first identify candidate pockets that have drug-like physicochemical properties but may lack sufficient drug-like size, and then seek out energetically accessible side-chain and backbone motions near these potential pockets using protein modeling approaches.

For determining whether a target pocket has drug-like physiochemical properties, we use an adaptation of a validated druggability score [13]-[15], which we call Dscore+. Dscore+ is a modification of Dscore [13], and we've found that this modification results in good correlation with ^19^F NMR hit-rates for five newer targets prosecuted at Amgen [14]. Dscore+ is computed from physiochemical descriptors generated from a program, SiteMap, and is a weighted sum with contributions from the degree of pocket enclosure (a surrogate for pocket curvature), pocket size, and the balance between hydrophobic and hydrophilic character in the binding site [13]. In this work, we further validate Dscore+ as a druggability score, and tune the pocket identification parameters in the program, SiteMap, to better identify pockets that may become more druggable with protein motion. In addition to assessing the physiochemical properties of the pocket, we assess reasonable drug-like size by considering the volume of the pocket.

**Figure 1:**
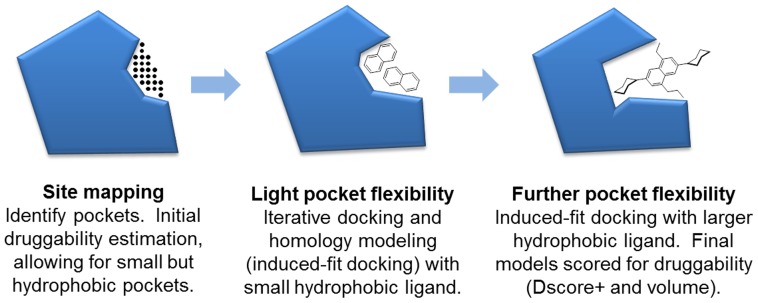
Method for druggability estimation with light protein flexibility. Multiple pockets per protein are considered, but one is shown here for simplicity.

The method consists of the three steps depicted in Figure 1. If a protein site is found to meet a minimal Dscore+ threshold, then residues surrounding the site are put through an iterative flexible protein docking and protein modeling workflow known as induced-fit docking [16] in order to model protein flexibility, including light, local backbone movement and side-chain rotamer conformations. Induced-fit docking is done twice, first using a small naphthalene molecule to reorder side-chains and expose small hydrophobic clefts, and then again using a larger, tetra-substituted naphthalene molecule to further open the cleft if the protein structure allows. We note that others have used fragment docking to assess druggability of pockets in static structures [17], but we are using docking for the different purpose of inducing flexibility in pockets.

We chose naphthalene and a larger tetra-substituted naphthalene because they are hydrophobic and aromatic—known features of drug-like molecules. Additionally, naphthalene is rigid so docking is fast. The tetra-substituted naphthalene molecule we use is a natural progression from naphthalene, and includes four substituents (ethyl, propyl, and cyclohexyl at two positions) that we thought could help in opening pockets. These simple-minded choices performed reasonably well in validation studies, and limited experimentation with a few other molecules gave similar or worse results. In particular, use of benzene in place of naphthalene resulted in a large false positive rate because benzene is small and much more promiscuous, fitting into many small sites. For the larger molecule, we tried five molecules similar to tetra-substituted naphthalene and the results were not substantially different. It is certainly plausible that more systematic experimentation with a larger number of ligands could result in improved performance.

Other approaches that address the issue of protein flexibility for druggability assessment use computational solvent mapping or molecular dynamics simulations, or both. *Kozakov et al.* used computational solvent mapping with 16 small organic fragment probes to identify small pocket ‘hot spots’ where multiple probes bind in the simulations [18]–[19]. Alternate side-chain conformers are then modeled for selected residues adjacent to the hot spots, and the resulting modeled sites are subjected to a round of computational solvent mapping. ‘Hot spots’ having all 16 probes bound are identified as druggable [19]. *Bakan et al.* used molecular dynamics simulations for solvent mapping with a different set of small fragment probes and found that the probes bind known allosteric sites during the simulation [20]. *Brown and Hajduk* earlier showed that molecular dynamics simulations can capture pocket dynamics that result in a more druggable binding pocket in Bcl-xl, while preserving much of the known binding site rigidity of Akt-PH and FKBP [21]. Tools have very recently become available to facilitate druggability assessment in molecular dynamics trajectories [22]–[24]. However, the typically-used short molecular dynamics simulations on the order of 10–30 ns likely do not fully capture protein flexibility relevant to drug binding [25], which typically occurs on much longer timescales [26].

Our approach to flexibly treating potentially druggable binding sites is substantially less compute-intensive, which is important for our goal of analyzing the structural proteome. For a single binding site where flexibility is modeled, our approach requires between one and two hours for most individual protein structures on a current scientific workstation with a four core CPU. In contrast, a 30 ns molecular dynamics simulation on a single protein would require about a week, and the computational solvent mapping approach using FTMAP requires about half a day for each protein binding site since FTMap must be run for each discrete side-chain configuration and each configuration requires about two hours on a single CPU core [27]. Our approach runs relatively quickly due to the use of comparative protein modeling techniques instead of more resource-intensive methods that attempt to directly simulate biophysical phenomena. Our approach finds very few sites that open up significantly—less than two percent of protein structures across the mammalian structural proteome. The false positive rate is reasonably low; we see a 12% false positive rate in our protein-protein interaction validation set and a 0% false negative rate. In contrast, it is likely possible to find all known druggable sites using molecular dynamics, including some that are undetectable by our method. However, separating the signal from the noise is challenging [20], [21] since there is a tendency for many pocket openings to be observed. To be clear, our approach does not reveal any new pockets that do not already exist within the rigid structure. It does, however, locate small sites that do not meet the druggability criteria initially but can meet the druggability criteria when conservative protein flexibility is modeled.

While accurate modeling of protein motion continues to be difficult and the subject of substantial research, we found the approach we present here to be sufficiently accurate and efficient for the purposes of mining the structural proteome. We note that previous efforts we are aware of to identify the “druggable genome” rely on sequence-similarity to known druggable proteins [2]. Structure-based druggability analyses are based on “first principles” and are thus complementary to precedence-based sequence-similarity approaches.

### Validation with targets of known druggability

We applied the method to two widely-used druggability validation sets to check its performance and measure any increase in false positive rate due to allowance of protein flexibility.

**Figure 2:**
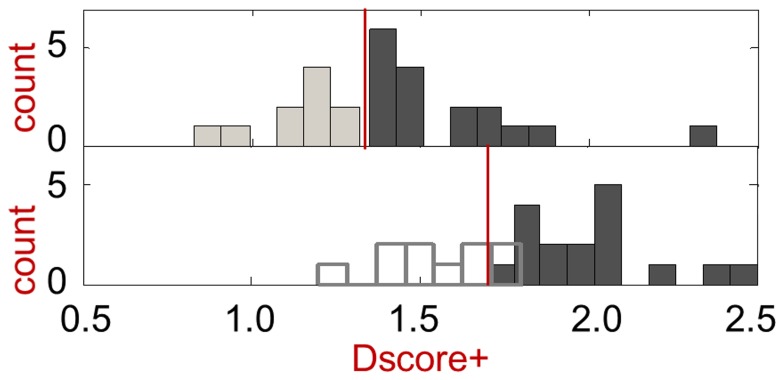
Druggability score histograms for validation set binding site structures. The top panel depicts results with the original crystal structures used rigidly, with a red line indicating the Dscore+>1.3 cutoff used in this work. The top Dscore+ value is shown for each of the 27 protein targets (17 druggable and 10 difficult, where the prodrug targets are considered difficult). The bottom panel depicts results after modeling of protein flexibility, with difficult targets in ghost outline because flexibility modeling is not usually applied to sites that score below the Dscore+>1.3 cutoff. Difficult targets are indicated by the lighter bars, while druggable targets are indicated by the darker bars. See text for further discussion.

The first validation set is a published set that covers a variety of targets, and consists of 27 targets: 17 druggable targets and 10 difficult targets [4]. A histogram of the druggability scores, Dscore+, based on the original crystal structures, with no flexibility modeling, is shown in Figure 2a. The plot supports a Dscore+ >1.3 threshold for druggable versus difficult targets, with higher scores roughly indicating more druggable sites. Modeling protein flexibility for target sites that meet a threshold of Dscore+>1.3 results in an increase in Dscore+, but the increase is relatively systematic (mean = 0.4, σ = 0.3) and appears to be consistent enough that a useful differentiation between difficult and druggable targets is retained.

We also investigated the effect of flexibility modeling on targets with scores of Dscore+≤1.3. For these additional targets, we again find an increase in scores by an average of 0.4 (σ = 0.3). Thus, difficult and druggable targets in the validation set can still be distinguished after flexibility modeling, although the distinction is less crisp than it was when scoring rigid structures. Comparing the score distributions for druggable and difficult targets using the two-sample Kolmogorov-Smirnov (K-S) statistic finds that the scores are significantly different from each other, both with and without flexibility modeling (*p*-values of 8×10^−4^ and 1×10^−6^, respectively). When we subtract 0.4 from each score that includes protein flexibility, the score distributions remain similar within each set. For druggable targets, the means of the with- and without-flexibility scores are 1.6 and 1.6 respectively, with variances of 0.05 and 0.07. For difficult targets, the means of the with- and without-flexibility scores are 1.2 and 1.1 respectively, with variances of 0.04 and 0.03.

Taken together, the results suggest that a Dscore+ threshold of 1.7 (i.e., 1.3+0.4) should be applied to sites resulting from flexibility modeling, and this threshold is depicted in Figure 2b by a red line. We will show that this threshold value is strongly supported by analysis of a larger number of proteins (109 proteins) from the mammalian proteome druggability results. While the threshold is determined empirically and the increase is not ideal, we can rationalize the increase as due to modeling of protein flexibility with an impetus towards making the pocket more hydrophobic.

Turning to pocket volumes, the method should not lead to all pockets increasing significantly in volume, consistent with the belief that some sites are inherently flexible while others are less so. In this first validation set, which is composed largely of enzyme active sites, the average volume before and after flexibility modeling are both about the same (420 Å^3^ and 360 Å^3^, respectively, with standard deviations of 190 Å^3^ and 130 Å^3^), and are both within the drug-like range, as discussed later. In the second, validation set of protein-protein interfaces, we will see that the binding site volumes change more significantly. Resulting volumes tend towards a volume of around 300–400 Å^3^, if the flexibility of the protein allows, and this appears to be related to the size of the second ligand (tetra-substituted naphthalene) used in the induced-fit docking step of the flexibility modeling. In the mammalian proteome analysis, we find that only one percent of proteins analyzed have cryptic pockets that change substantially from a volume substantially below the drug-like range (≤100 Å^3^) to a drug-like volume (160–800 Å^3^). In developing our approach, the drug-like volume range was initially set roughly to 150–600 Å^3^ based on our judgment, and later refined to 160–800 Å^3^ based on quantitative analysis of the mammalian proteome results.

### Validation with protein-protein interaction targets

The second validation set addresses protein-protein interaction (PPI) targets, and includes six targets from the 2P2I database and Wells et al. (2007): Bcl-xL, HDM2, IL-2R, HPV E2, ZipA, TNFα [10], [12]. Bcl-xL and HDM2 are classified as druggable since oral small molecule inhibitors have advanced to clinical trials. We argue that the remaining potentially high-value targets are difficult. While pioneering small-molecule inhibitors have been reported, we note that substantial efforts made over the last 15 years to identify inhibitors of TNFα, IL-2R, HPV E2, and ZipA have not resulted in reported small molecule clinical compounds [10]. Given just the protein crystal structures, with no information on location of binding sites, the method successfully opens the relevant binding pockets for Bcl-xL, HDM2, and TNFα and scores them as druggable based on Dscore+ and volume considerations, as shown in Table 1. Calculated values of Dscore+ and volume that fall within the defined drug-like range are highlighted in bold. In the cases of Bcl-xL and TNFα, light flexibility results in small molecule binding pockets with roughly 50% and 100% larger volumes, respectively. Bcl-xL would have been classified as difficult based on the 2bzw PDB structure without additional flexibility modeling because the pocket size (112 Å^3^) is too small by any reasonable criteria for drug-like volume size. Flexibility modeling results in small changes in the binding site that, together, increases the volume of the pocket to a reasonable volume (172 Å^3^). Bcl-xL is perhaps the one clear example where a protein pocket opens substantially and the druggability is known (i.e., orally administered small molecule inhibitors have progressed to clinic).

**Table 1:** Results for protein-protein interaction targets from 2P2PI with known druggability [10], [12].

Target name	PDB ID	Ligand type	Assigned druggability	Crystal pocket		Flexible model	
				Dscore+	Volume	Dscore+	Volume
Bcl-xL	2bzw	protein	druggable	**1.5**	112	**2.4**	**172**
Bcl-2	2xa0	protein	druggable	**1.5**	**167**	**2.0**	**174**
HDM2	1ycr	protein	druggable	**1.7**	**165**	**2.5**	**175**
TNFα	1tnf	protein	difficult	**1.8**	126	**2.4**	**257**
IL-2Rα	1z92	protein	difficult	0.9	49	*	*
HPV E2	1tue	protein	difficult	0.8	57	*	*
ZipA	1f46	protein	difficult	0.7	105	*	*
ZipA	1f47	protein	difficult	0.9	141	*	*
Bcl-xL	2yxj	cmpd	druggable	**1.8**	141	**2.5**	**239**
Bcl-xL	3qkd	cmpd	druggable	**1.9**	132	**2.1**	**195**
Bcl-xL	4ehr	cmpd	druggable	**1.4**	100	**2.4**	**175**
Bcl-2	4aq3	cmpd	druggable	**1.7**	113	**2.3**	**220**
HDM2	1rv1	cmpd	druggable	**1.7**	147	**2.2**	**178**
HDM2	1t4e	cmpd	druggable	**1.6**	**203**	**2.1**	**234**
HDM2	3jzk	cmpd	druggable	**1.6**	**204**	**1.9**	**184**
HDM2	3lbk	cmpd	druggable	**1.5**	150	**1.9**	**160**
HDM2	3lbl	cmpd	druggable	**2.0**	**224**	**2.2**	**218**
HDM2	3tu1	cmpd	druggable	**1.5**	**221**	**1.9**	**172**
HDM2	4dij	cmpd	druggable	**1.6**	**192**	**1.8**	**207**
HDM2	4ere	cmpd	druggable	**1.8**	**165**	**2.5**	**172**
TNFα	2az5	cmpd	difficult	**1.7**	**325**	**2.0**	**233**
TNFR1	1ft4	cmpd	difficult	1.2	**330**	*	*
IL-2Rα	1py2	cmpd	difficult	1.2	66	*	*
IL-2Rα	1pw6	cmpd	difficult	**1.4**	73	**1.4**	72
HPV E2	1r6n	cmpd	difficult	1.2	95	*	*
ZipA	1y2f	cmpd	difficult	0.9	91	*	*
ZipA	1y2g	cmpd	difficult	0.9	96	*	*

These targets have protein-protein co-crystal structures shown in the top half of the table, and corresponding protein-ligand co-crystal structures are shown in the bottom half. Values of Dscore+ and volumes falling within drug-like criteria are highlighted in bold. ‘cmpd’ indicates a small molecule compound, and volumes are in units of Å^3^. “*” indicates ‘not applicable’ because the site's initial Dscore+ values did not meet the cut-off for flexibility modeling; however, some values were subsequently calculated for validation purposes, and are discussed in the text. We define known druggability based on the success in finding drug-like, clinical small molecules where there are efforts from multiple independent groups.

We also analyzed all targets listed in 2P2I where crystal structures are provided, but some targets have either unclear experimental druggability because efforts on the targets are more recent, or known inhibitors involve metal chelation. The results for these additional targets are included in Table S1.

Comparing Dscore+ and pocket volume calculation results with and without protein flexibility modeling finds that Bcl-xL (PDB IDs: 2bzw, 2yxj, 3qkd, 4ehr) and a minority of HDM2 structures (PDB IDs: 1rv1, 3lbk) would have been missed without the additional flexibility modeling to open up pockets to a drug-like volume. Interestingly, one IL-2Rα structure (PDB ID: 1pw6) has a Dscore+ that places the target in the low end of the druggable score range, but the pocket volume does not satisfy the drug-like criteria, and this remains the case after protein flexibility modeling. Protein flexibility modeling does not always open pockets significantly.

With TNFα, the known pocket at the trimer interface was identified as the top pocket in the apo-structure, and flexibility modeling resulted in a binding site with good druggability score and good drug-like volume. This result is consistent with the scores obtained using the co-complex structure with SPD-304 [28]. However, the best reported inhibitor has only single digit micromolar range potency against TNFα [28], and although there is not really sufficient data currently to determine this, it is possible that the calculations overestimate the druggability of the pocket.

**Figure 3:**
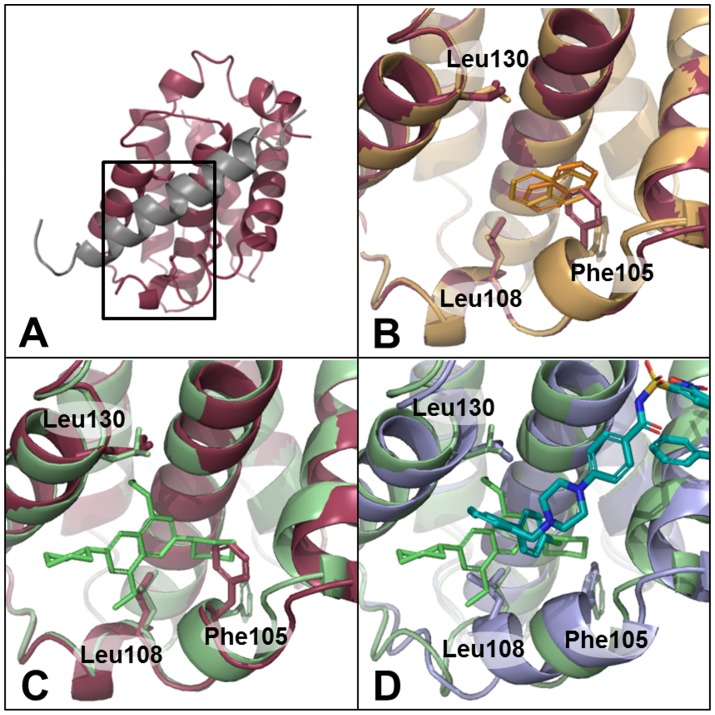
Flexible protein druggability modeling applied to Bcl-xL. Phe105, Leu108, and Leu130 are shown in stick in all structures. (a) Crystal structure of Bcl-xl protein bound to a BAD peptide (red and gray, respectively, PDB ID: 2bzw), (b) two naphthalene induced-fit docked models (orange), (c) one TSN induced-fit docked model (green), and (d) ABT-737-bound crystal structure (blue, PDB ID: 2yxj) with TSN induced-fit model (green).

With Bcl-xL, comparison of a BAD peptide-bound structure (PDB ID: 2bzw) with a small molecule-bound structure (PDB ID: 2yxj) shows that two residues, Phe105 and Leu130, adopt alternate conformations, and the helix around Leu108 becomes disordered to create the ligand binding pocket [29], as shown in Figure 3. The target serves as a good illustrative example of our complete approach. First, a potentially druggable site is identified regardless of whether it is too small to hold a drug-like molecule. This is followed by induced-fit docking of naphthalene to the identified site, which moves residues, including Phe105, as shown in Figure 3b. A second induced-fit docking of the larger (molecular weight of 363 Da) tetra-substituted naphthalene (TSN) results in a total of four models, where we see additional movements in addition to Phe105. A representative model is shown in green in Figure 3c, and shows a Leu130 rotamer change and backbone movements around Leu108 resulting in loss of the alpha-helical secondary structure. However, the modeled structures still differ from the ligand-bound crystal structure, as shown in Figure 3d, and the model, in this case, is effectively a hybrid of the peptide-bound structure and the known ligand-bound structure. Thus, the modeling approach, in the case of Bcl-xL, successfully allows backbone motion and reproduces some of the known side-chain and backbone movements in the resultant models. We note that the TSN molecule makes similar interactions compared to the ligand, ABT-737, in the ligand-bound crystal structure. Despite not reproducing all of the atomic details of the ABT-737 crystal structure, the flexibility modeling captures many key features and the inherent flexibility of the pocket that results in an increased binding site volume and increased druggability score.

### Known protein flexibility cases

To investigate the behavior of the flexibility modeling approach on targets where protein flexibility is known based on crystal structures, we applied the method to a set of protein crystal structures with binding site flexibility from *Huang and Jacobson* [17], where we've selected targets that show RMSD_ave_ >1.5 Å for binding site residues in different crystal structures of the same protein. We compare these results with their published druggability results, which do not account for flexibility, in Table 2. While the two methods perform similarly on non-PPI targets (first six targets in Table 2), the flexible druggability method performs better on PPI targets (last five targets in Table 2). In particular, their docking-based druggability model predicts IL-2 and HPV E2 to be druggable (docking hit rate>0.36) based on some structures, but predicts the same targets to be very difficult based on other structures. The flexibility modeling approach results in classification of both targets as very difficult, consistent with what is known, as previously discussed.

**Table 2:** Comparison of druggability estimations on known flexible protein pockets.

Target	Structural data		Docking-based druggability	Protein flexibility	Variation	
	PDB ID	RMSD_ave_ (Å)	[A] dock hit rate	[B] DScore+	[A]	[B]
CDK2	1aq1		1.32	1.7	21%	11%
	1buh	1.8	1.44	1.7		
	1dm2	1.8	1.62	1.9		
ER	1l2i		1.69	2.9	9%	7%
	3ert	2.6	1.55	2.7		
	1err	2.0	1.61	2.8		
HIV RT	1vrt		1.66	2.5	8%	13%
	1rt1	1.5	1.75	2.3		
	1c1c	1.9	1.61	2.2		
	1rth	1.6	1.61	2.3		
p38α	1a9u		1.00	1.8	49%	15%
kinase	1kv1	3.8	1.16	2.1		
	1kv2	3.5	1.61	2.1		
PPARγ	1fm6		1.46	2.9	13%	34%
	1fm9	1.5	1.62	3.0		
	2prg	0.7	1.43	2.1		
TK	1kim		1.58	2.7	12%	4%
	1ki4	1.8	1.40	2.6		
IL-2	1z92		0.13	*	107%	*13%*
	1py2	2.6	0.62	*		
	1m48	2.5	0.62	*		
Bcl-XL	2bzw		1.04	2.4	21%	4%
	2yxj	2.5	0.84	2.5		
TNF	1tnf		0.95	2.4	1%	18%
	2az5	2.9	0.96	2.0		
MDM2	1ycr		0.45	2.5	69%	18%
	1rv1	1.8	0.92	2.2		
	1t4e	1.6	0.66	2.1		
HPV E2	1tue		-0.24	*	323%	*31%*
	1r6n	2.8	1.02	*		

Targets are from *Huang and Jacobson* [17], and include all targets where at least two structures have an RMSD_ave_ greater than 1.5 Å. The data under the *Structural data* and *Docking-based druggability* are from reference 17. RMSD_ave_ is the RMSD of side-chains in the binding site within 4.5 Å of crystallographic ligands.[17] *Variation* is calculated as the difference between the largest and smallest druggability score values divided by the average of all druggability score values for the particular target. The flexible druggability method is only performed for binding sites that meet an initial score (with the rigid crystal structure). However, for the purposes of this comparison study, we removed this cut-off in order to generate values for IL-2 and HPV E2. For IL-2, performing the flexibility modeling procedure results in DScore+ values of 1.5 (1z92), 1.5 (1py2), and 1.7 (1m48), with small, non-drug-like volumes of 98, 82, and 53 Å^3^, respectively. For HPV E2, the DScore+ values are 1.1 (1tue) and 1.5 (1r6n), with reasonable drug-like volumes. Calculations on all targets from reference 17 are included as S4.

Examining the variation in scores between different crystal structures of the same target finds that while both the static protein and flexible protein methods yield similar score variation for non-PPI targets, they have substantially different variation with PPI targets. In particular, the docking hit-rate method shows large variation in score among structures of IL-2 (107%), MDM2 (69%), and HPV E2 (323%) compared with a median variation of 21% in all 11 targets. The flexibility modeling method, on the other hand, results in score variation on PPI targets that is consistent with that found with non-PPI targets.

Overall, the docking method has a median score variation of 21% with a standard deviation of 94% in the dataset, while the flexibility modeling approach has a median score variation of 13% with a standard deviation of 10%. Yet, when the PPI targets are removed, the two methods have comparable score variation. Taken together, the flexibility modeling method appear to provide more reliable, consistent predictions at PPI interfaces, and this makes sense because PPI interfaces are much more likely to involve substantial protein flexibility [11]. Accounting for protein flexibility in a conservative manner, as we have done, leads to more consistent druggability predictions.

### The mammalian structural proteome

We next applied the flexibility modeling approach to all publicly-available crystal structures containing mammalian proteins to estimate the number of druggable targets and identify potential druggable cryptic pockets. Analyzing the over 18,000 structures in the Protein Data Bank (PDB) [30] as of June 30, 2012, required approximately 35,000 CPU-days of calculation (in aggregate) on our internal Linux clusters. The analysis covered not only the crystal structures as deposited in the PDB, but also all individual monomers in the case of multimer assemblies. Five percent of PDB files generated an error, due mostly to structures containing only Cα atoms (i.e., no protein side-chains) or involving large biomolecular assemblies, since we stopped calculations on a particular PDB entry if it ran for more than fourteen days.

The results are summarized in Table 3, where we also mapped PDB chains to SWISS-PROT ID's to determine the number of proteins represented. Of the 17,834 PDB entries analyzed, 42% had at least one site that met the Dscore+ >1.3 threshold for further protein flexibility modeling. Twenty percent of mammalian proteins in the PDB have a potentially druggable pocket. Of the 5,739 PDB entries (1,134 proteins) that have a predicted druggable pocket, about two–thirds would be predicted druggable based on the original, static crystal structure.

**Table 3:** Results of druggability analysis of mammalian crystal structures in the PDB (as of June 30, 2012) with inclusion of light protein flexibility.

	Structures	Proteins	
mammalian crystal structures	18,879	5,807	105%
structures analyzed	17,834	5,551	100%
flexibility modeling applied 1	7,427	2,875	52%
potentially druggable 2	5,739	1,134	20%
involves intermolecular interface^3^	2,095	730	17%
cryptic pocket^4^	105	69	1%

The “Structures” column provides the number of unique PDB entries represented, and “Proteins” represents the number of unique Swiss-Prot entries.

1 Dscore+>1.3 for original rigid structure.

2 Dscore+≥1.7, drug-like volume (160–800 Å^3^) after flexibility modeling, protein at least 100 amino acids in size (equivalent to about 10 kDa molecular weight).

3 Intermolecular interfaces are further defined to include only protein-protein interaction dimer interfaces and protein-ligand pockets.

4 Cryptic pockets are further defined as pockets that are less than 100 Å^3^ in volume in the crystal structure, but fall into the drug-like volume range after modeling of protein flexibility. An additional criteria of enclosure <96% was applied to eliminate small buried sites.

To identify druggable pockets with the greatest likelihood of biological relevance, we winnowed the list to protein sites in 2095 PDB structures where a small molecule could potentially disrupt a known intermolecular interaction. The interacting partner should be transiently-bound (as opposed to obligately-bound) and can be a protein, natural co-factor, natural ligand, or synthetic ligand. These sites are either at protein-protein interfaces or contain a small molecule of molecular weight less than 1000 Da. Including these criteria gives us higher-confidence druggability predictions and may remove many false-positives, but could result in removing sites that are functionally relevant but perhaps not well-characterized. For sites at protein-protein binding interfaces, we assessed whether the relevant protein-protein interaction is an obligate or transient interaction based on a published database, Interevol [31]. Sites involving obligate dimer interfaces were removed, but sites without any prediction or assessment were retained; there was no annotation for over half of the protein interfaces we considered.

**Figure 4:**
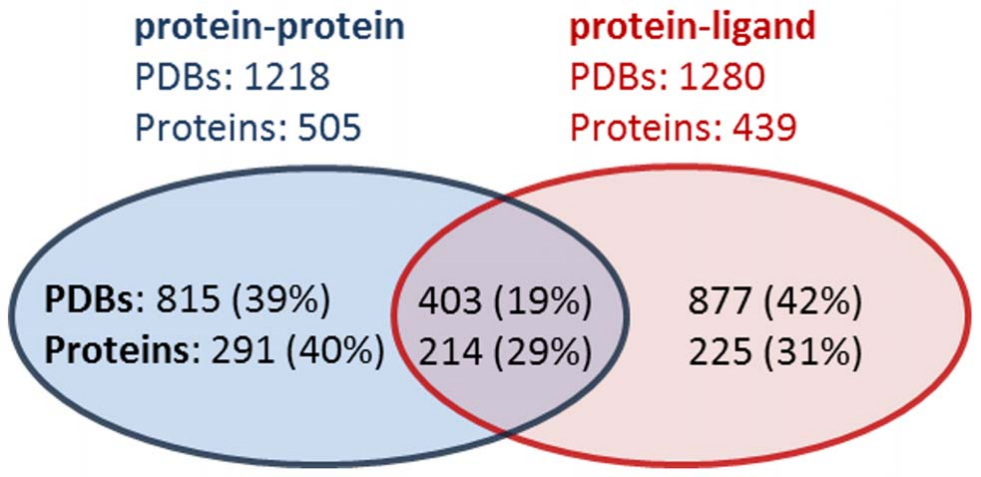
Reasonable druggable criteria from analysis of 109 co-crystal structures of MDDR oral tablet drugs. A) Volumes of MDDR protein structure pockets. B) For pockets with volumes between 160 and 800 Å^3^, Dscore+ distribution of MDDR protein structures (top) and all protein structures (bottom). C) Range of Dscore+ after flexibility modeling of MDDR protein structure pockets supports a modified 1.7 Dscore+ cut-off after flexibility is applied.

Overall, we identified predicted druggable pockets in 2,095 PDB structures representing 730 unique proteins. In Figure 4, we depict the breakdown of predicted druggable pockets at these intermolecular interfaces with pockets where a bound ligand would disrupt a protein-protein interaction (including protein-peptide interactions) shown in blue, and pockets where a bound ligand would disrupt a protein-ligand interaction shown in red. The purple overlap region indicates protein pockets that are at both a protein-ligand and protein-protein interface. Some of these pockets are adjacent to small peptides, which can be classified as both a ligand and protein by our definition; ligands are defined as any molecule with molecular weight of 1000 kDa or less, while proteins are defined as non-HETATM molecules. The number of unique proteins in the purple region is much higher because a given protein may not only have a co-crystal structure solved bound to a protein partner, but also bound to a small molecule ligand (or vice-versa).

To identify cryptic pockets, we looked at potential druggable pockets that were small (volume ≤100 Å^3^) in the static structure, as long as the initial cavity was not fully buried (enclosure ≤96%). Less than 20% of these, representing 105 structures, met the flexible druggability criteria, opening up to at least 160 Å^3^ with flexibility modeling. These targets representing 69 unique proteins are provided in Table S2.

**Figure 5:**
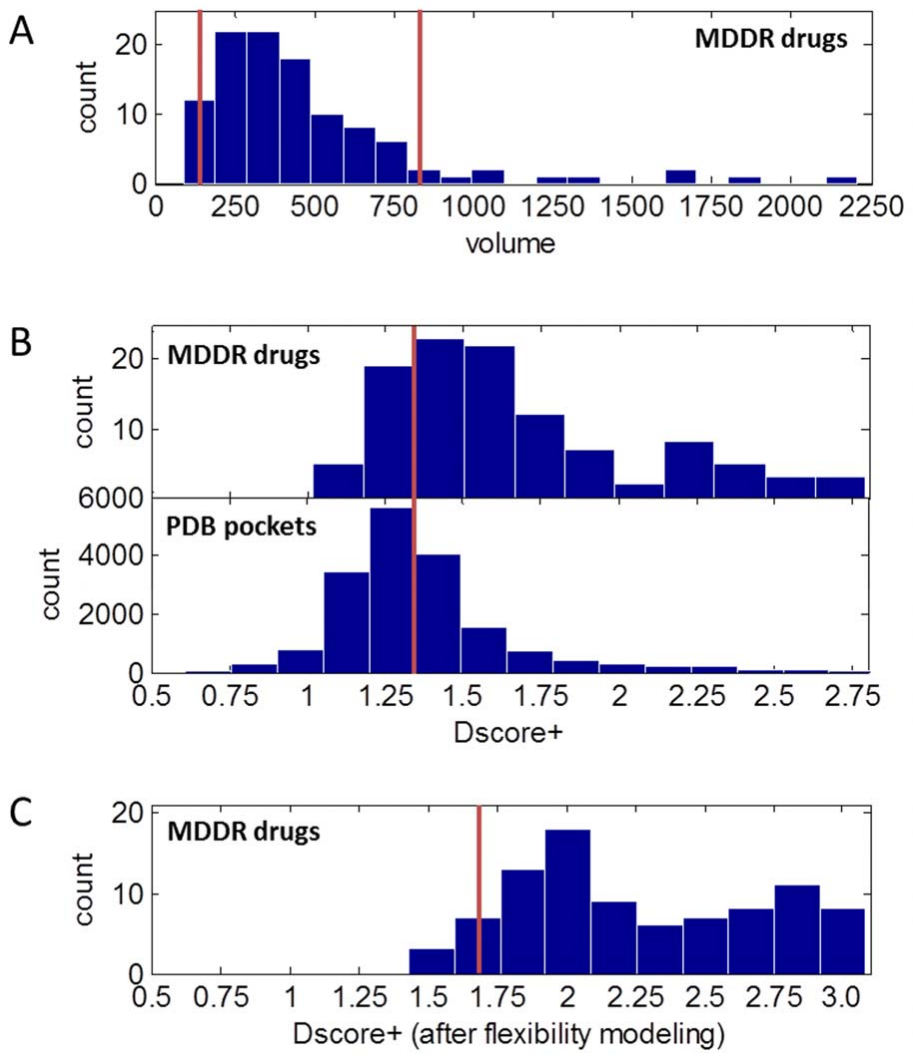
Flexibility modeling substantially increases the volume of a minority of protein pockets. A 2D histogram showing all pockets found in the mammalian structural proteome with initial volumes less than 800 Å^3^ and with greater than 100 amino acids (about 10 kD in weight). The vertical and horizontal white lines indicate the 160 Å^3^ volume cut-off. While the modeling method likely overpredicts volume increases in pockets, the majority of pockets that increase in volume increase by less than 50 Å^3^. The color bar on the right side indicates the number of pockets at each 2D histogram bin.

To compare the mammalian PDB results to a positive control set, we mapped known oral drugs from MDDR (2008 release) to ligands in known crystal structures. Of the 421 oral drugs administered in tablet form, 109 could be mapped to PDB co-crystal structures that had crystallographic resolution ≤2.5A. The 102 pockets with ligand overlap to known co-crystalized ligands (ligand overlap >0) are plotted by volume in Figure 5A, where volume is computed using SiteMap. Targets at the low end of the volume range, with volumes of 160 Å^3^ or less, include four complexes with large FK-506 natural product analogs that are not captured by the drug-like binding site definition in use. Targets with volumes of 800 Å^3^ or greater are largely natural product complexes as well (macrocyclic antibiotics, reservatrol, and others). The results therefore suggest a drug-like volume range of between 160 and 800 Å^3^ is appropriate for the approach we use. We note that volume calculations are highly sensitive to the algorithm used, and so these volume ranges should be established independently for different implementations of our approach.

The range of druggability scores for the known oral tablet drug set versus all pockets is shown in Figure 5B, where the top histogram represents the oral tablet drug set. The distributions are overlapping, and while the means of 1.7 and 1.4 are significantly different (p = 6×10^−15^ based on the two-sample K-S test), the 95% confidence intervals overlap. While the large-scale data shows there is room for improvement in the separation of druggable and difficult targets, the 1.3 Dscore+ cut-off we use is nevertheless useful for identifying druggable pockets in rigid proteins, and removing 60% of pockets from further analysis with more resource-intensive flexibility modeling. Applying flexibility modeling to the MDDR targets also results in a shift in Dscore+ range, shown in Figure 5C, similar to what is seen with the smaller general validation set. The shift seen here further supports use of a Dscore+>1.7 cut-off in conjunction with protein flexibility modeling.

**Figure 6:**
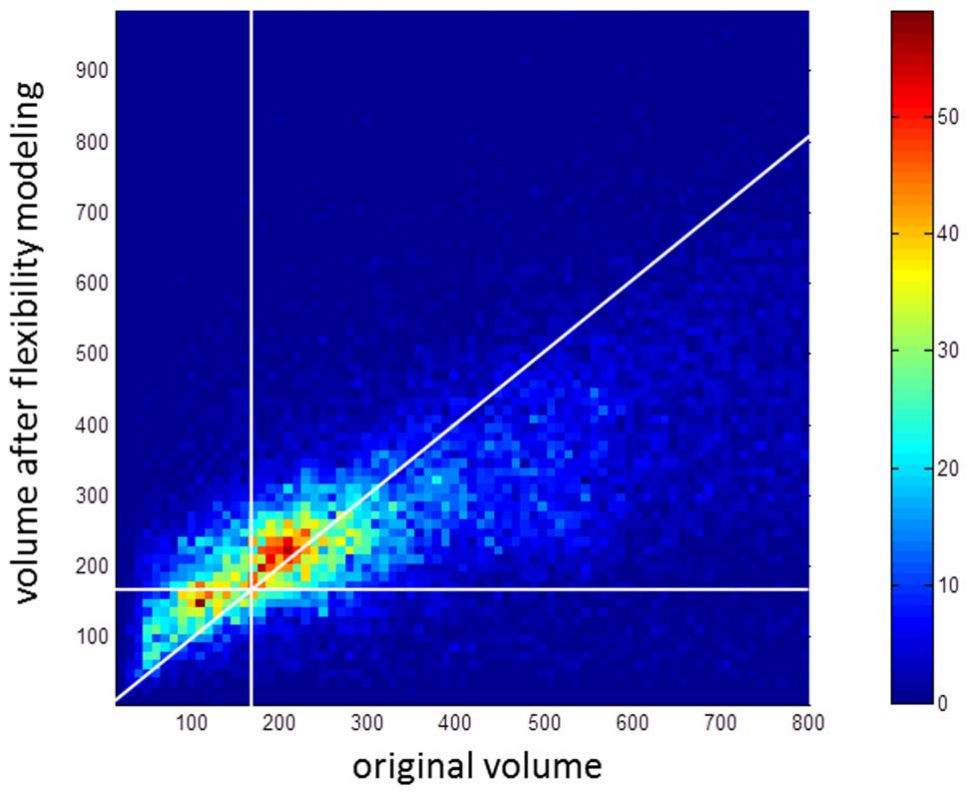
Breakdown of predicted druggable pockets at known intermolecular interfaces. Pockets found where a bound ligand would disrupt a protein-protein interaction are shown in the blue circle, and pockets found where a bound ligand would disrupt a protein-ligand interaction are shown in red. The overlap region shown in purple indicates where a protein or structure contains a pocket at both a protein-ligand and protein-protein interface. The blue region indicates proteins or structures containing only protein-protein interfacial pockets, and the red region indicates proteins or structures containing only protein-ligand pockets. Ligands are defined as any molecule with molecular weight ≤1000 kDa.

To assess the effect of our flexibility modeling approach on pocket volumes, we looked at all pockets at intermolecular interfaces before and after flexibility modeling and show the results in Figure 6. A diagonal white line indicates no change in volume. While the modeling method likely overpredicts volume increases in pockets, the majority of pockets that increase in volume increase by less than 50 Å^3^. The vertical and horizontal white lines in Figure 6 indicate the 160 Å^3^ volume cut-off, and it is clear that most pockets under the cut-off remain under the cut-off. As expected, pockets with volumes closer to the cut-off, with volumes of 100–160 Å^3^, are the most likely to increase to over 160 Å^3^ with protein flexibility modeling.

In Figure 6, pockets with original volumes less than about 200 Å^3^ tend to get larger, while pockets with original volumes greater than about 400 Å^3^ tend to get smaller. The likely rationale is that the tetra-substituted naphthalene ligand used in the flexibility modeling approach induces smaller pockets to grow to accommodate the ligand, while it induces larger pockets to shrink to better enshroud the ligand. These tendencies are, however, dependent on the inherent flexibility of the protein structure.

While the analysis provides a good set of putative druggable proteins in the mammalian structural proteome, we are not blind to deficiencies in this analysis. The prediction error rate in the large mammalian structural proteome analysis is hard to know, and we discuss the limitations in the next section.

### Limitations and future work

The automated approach to protein flexibility we report here is useful for identifying druggable targets in the structural proteome. We are aware of three areas for further improvement.

**Figure 7:**
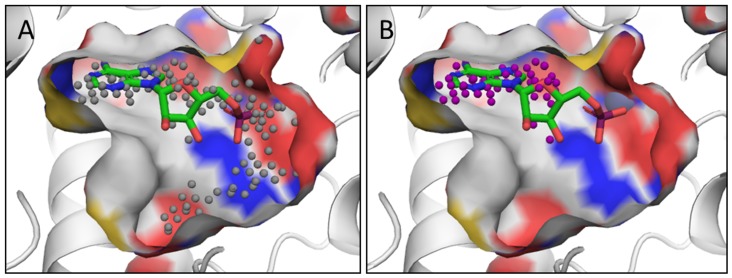
Example of druggability predictions PDE-4D binding site (PDB ID: 1ptw) druggability calculations: (a) gray spheres defining the full automatically identified site, (b) purple spheres depicting the edited subsite. The crystal structure is of AMP-bound PDE-4D.

The first is related to pocket selection. Pocket selection is based on geometric considerations, and the pockets are subsequently scored for druggability using Dscore+ as well as, potentially, protein flexibility modeling. Ideally, the pocket selection and scoring would be done simultaneously to yield pockets that maximized the druggability score [32]. This issue, for instance, has an effect on scoring of phosphodiesterase active sites such as those in PDE-4D and PDE-5, where protein residues at one end of the catalytic site are very polar, and known oral inhibitors do not interact in this region [32]. Figure 7a shows the binding site including the polar region that results in a low druggability score, Dscore+ = 1.4, which is not representative of the druggability of the binding site. Removing the polar region shown in Figure 7b results in a more representative druggability score, Dscore+ = 1.6. We were not successful in adjusting our protocol to account for this, and thus we may miss druggable sites that are similarly amphipathic in nature. In addition, despite our efforts at tuning the pocket identification algorithm, SiteMap does, in about 2% of cases, return candidate pockets with volumes over 800 Å^3^, the drug-like size limit that we use. Currently we simply remove these pockets, which may cause us to miss druggable sites. Future work to resolve these issues include modifying SiteMap to identify only pockets of the desired volume (160–800 Å^3^), allow for pockets that overlap with each other, and account for properties such as hydrophobicity in the pocket definition process.

A second area relates to the false positive rate, that is, the fraction of pockets identified as druggable that are not truly druggable. Even though we restrict protein flexibility to side-chain motion and localized backbone movement, the protein flexibility modeled and our selection of proble molecules are biased towards increasing the hydrophobicity of the pocket under analysis, and relaxation of the resultant structures may improve results. In addition, the degree of protein flexibility modeled is probably more than that present in reality. In this work, we empirically compensated for these issues by measuring the impact of flexibility modeling in Figures 2 and 4, which led to the use of a Dscore+≥1.7 criteria. While our flexibility modeling approach demonstrates statistically significant discrimination of difficult and druggable targets, we also plan to explore approaches such as protein relaxation [33], to remove the need for an empirical correction. The flexibility modeling approach is more likely to exaggerate the flexibility of smaller proteins due to fewer stabilizing interactions within the protein. For the cryptic pocket analysis, we restricted our results to those proteins that are greater than 100 amino acids in length (which translates to about 10 kD).

Lastly, we need to consider that the protein structures observed in crystal structures, in a minority of cases, may not be the biologically relevant constructs or complexes. Crystal structures may be synthetic constructs or portions of proteins, which, in the context of the full-length protein, have predicted binding sites occluded. Similarly, biological obligate dimers not seen in the crystal structures can occlude the binding site. Co-factors can also affect the druggability of binding sites; here, we only account for selected, particularly tight-binding co-factors such as metals and hemes. We analyze both biological assemblies defined in the PDB as well as the individual monomer components to account for binding to intact complexes as well as unbound partners. Other partially dissociated complexes may exist however. In addition, we are looking at binding, and not functional effects of binding; weak binding at an allosteric site is sometimes sufficient to generate the desired inhibition or activation of biological activity [32].

### Conclusions

We leverage advances in druggability assessment and modeling of protein flexibility to create an approach that allows light flexibility in the protein backbone and side-chains. The method improves the accuracy of druggability assessments when tested on two validation sets representing general pharmaceutical targets and protein-protein interactions of pharmaceutical interest. Combining this with the wealth of crystal structures available in the PDB allows us to find new protein binding sites that are potentially druggable by small molecules. Searching for such sites is thought to be like finding needles in a large haystack, and a systematic, automated approach is thus useful. Accurate modeling of protein flexibility continues to be difficult and the subject of substantial research. Even so, our approach is useful in exploring induced druggable pockets and provides a substantial number of hypotheses. For applications focused on analysis of protein pockets, the approach we take is computationally efficient and may be complementary to comprehensive analyses of static crystal structures [34]. Finally, we have long been intrigued by the possibility of combining the druggability data with biological target disease-relevance data. This has recently been done on a small-scale with cancer targets [35], although protein flexibility was not accounted for in the druggability assessment. Inclusion of protein flexibility in such assessments can help to provide more accurate target assessment.

## Materials and Methods

Protein structures were downloaded from the biounits repository at the RCSB based on criteria that the structure (1) contains protein, (2) is categorized as deriving from the class *Mammalia*, and (3) has an x-ray crystal structure resolution ≤2.5A.

Ligands, defined as having molecular weight ≤1000 Da, are removed with the exception of heme groups, zinc, and magnesium (PDB het groups HEM, MHM, HEV, VER, SRM, HEO, HEB, HEC, HDM, HDD, DDH, ZN, MG). Protein structures were prepared using Schrödinger Protein Preparation Wizard (version 2012, Schrödinger LLC, New York, NY), on the command line with the following options: –watdist 0, –fillsidechains, –rehtreat, –mse, –noepik, –noimpref. These options assign bond orders, add hydrogens, remove all waters, create zero-order bonds to metals, create disulfide bonds for close cysteines, mutate selenomethionines to methionine, fill in any missing side-chains with Prime (v3.1, Schrödinger LLC, New York, NY), and optimize hydrogen placement and polar residue flips using PropKa. Validation test runs using restrained minimization to a heavy atom RMSD of 0.3 Å, a procedure known as “Impref”, did not change which sites were found and did not significantly change druggability scores on the validation dataset proteins, so we chose to increase workflow speed by avoiding this step in the protein preparation.

Next, initial potential druggable surface patches were identified using Schrödinger SiteMap (v2.6, Schrödinger LLC, New York, NY), the results of which are used to compute Dscore+. We run Sitemap with a fine grid (0.35 Å spacing) and “loose” definition of hydrophobicity. In this study, all calculations were performed from the command line with options that return the 5 largest SiteMap sites, in order of the number of site points they contain. Our modified settings allow more shallow binding sites to be found and include binding site regions with slightly weaker vdW interaction energy. We used the following non-default Sitemap parameters: maxdist = 10, enclosure = 0.4, maxvdw = 1.0, dthresh = 5.0, mingroup = 7, nthresh = 7, grid = 0.35, modphobic = 0. The smaller value of maxvdw (default is 1.1 kcal/mol) and the less restrictive definition for modphobic of zero together allow gridpoints with slightly weaker vdW interaction energy to be included as sitepoints. The smaller enclosure score (default is 0.5) and larger maxdist value (default 8.0 Å) allow more shallow binding sites to be found. The enclosure score is computed by drawing radial rays from each sitepoint, and the score is the fraction of rays that strike the receptor surface within a distance of 10 Å (maxdist), averaged over the sitepoints. Decreasing dthresh from the default (6.5 Å) and increasing nthresh from the default (3) causes SiteMap to return smaller, more compact sites than it otherwise would when using a fine grid. When considering a gridpoint for inclusion in a site, there must be at least nthresh other points within 1.76 Å (square root of d2thresh) for it to be considered. When considering whether two sites should be joined, the closest points in the two sites must be closer than dthresh. The parameter, “mingroup”, is the only parameter here that limits the number of sites found; this is the minimum number of points in a site-point group required to constitute a site (default = 7). We found that including sites with less than seven points in combination with a fine grid of 0.35A resulted in merging of many very small pockets to form long, stringy sites that were not realistic as small molecule binding sites. Overall, these modified SiteMap settings allow us to find shallow pockets with less hydrophobic character than is possible to find with default settings.

From the SiteMap results, sites identified with a druggability score, Dscore+, of greater than 1.3 are taken as candidate sites regardless of volume, where Dscore+ is defined as Dscore + 0.3*hydrophobic, as previously described [14], [15], and druggability scores are rounded to the first decimal place. The choice for the 1.3 value is discussed in the *Results and Discussion* section. Dscore is computed from physiochemical descriptors generated by SiteMap, and is a weighted sum with contributions from three components, (1) degree of site enclosure, (2) pocket size defined by the number of site points included in the site; site points are x, y, z coordinates that are outside the protein, are reasonably enclosed, and have a vdW interaction potential over a defined threshold are clustered into sites, and (3) a negative contribution from the hydrophilic score, which limits the impact of hydrophilicity in charged and highly polar sites [13].

**Figure 8:**
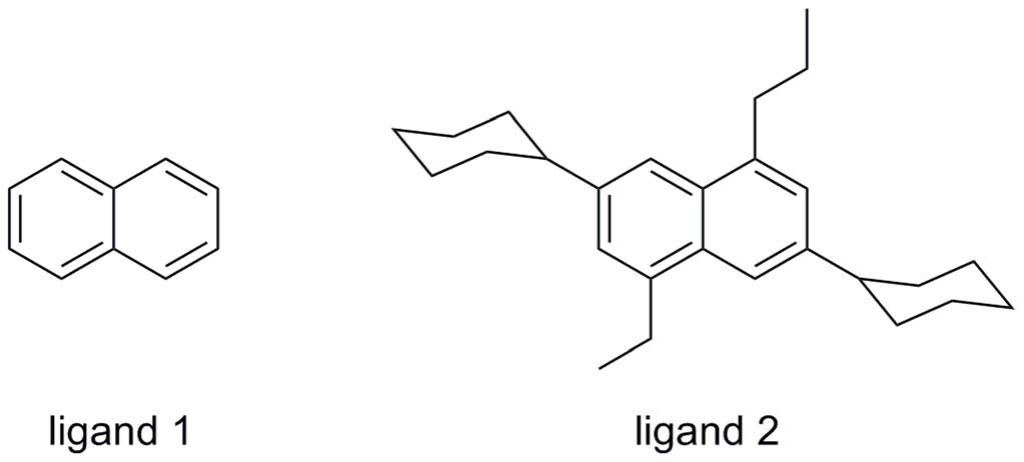
Ligands used in protein modeling and docking procedure. The tetra-substituted naphthalene compound, 2, was designed to facilitate opening of pockets.

To identify binding sites with potential flexibility, we used an iterative protein-modeling and docking approach [16], available in the Schrödinger Suite (2012 release, Schrödinger LLC, New York, NY) as the induced fit docking (IFD) workflow, and applied this using the ligands in Figure 8 to each candidate site. In this study, all calculations were performed from the command line with the default IFD parameters, except the variable, OUTER_BOX, which is always set to 25 Å, since we were docking the same small ligands. We defined the variable, BINDING_SITE, by a single sitepoint which is placed at the centroid of all SiteMap sitepoints from the candidate site. First, we used IFD to dock a naphthalene molecule, **1**, to the top 5 sites found by SiteMap and kept the best two naphthalene poses for each site. If poses were returned for naphthalene, we then used IFD again to dock a tetra-substituted naphthalene molecule, **2**, to the same pocket, now opened up by naphthalene. SiteMap was then applied to score the sites in the four top-scoring structural model results (typically, at least ten models were generated per site). Increasing the number of models can result in better predictions of binding site conformations, but we chose to produce a smaller but reasonable set of four models to reduce the compute time required to process all mammalian crystal structures.

To analyze the druggability and protein-protein interaction validation data, we automatically compared each SiteMap site to the corresponding ligand-bound structure using the Phase (version 3.4, Schrödinger LLC, New York, NY) command-line utility phase_volcalc to compute the overlap (measured in Å^3^) between the SiteMap sitepoints and the bound crystal ligand. After the IFD steps, we used the same utility to compute the overlap between the tetra-substituted naphthalene and the bound crystal ligand. This value is positive when there is direct overlap between the two sets of atoms. For the validation studies only, we identified the relevant protein biological assembly based on the known literature, and only retained those assemblies or protein monomers that are biologically meaningful. The calculations were otherwise performed automatically.

For calculations run on all mammalian PDB structures, we used a purely automated procedure applying the method to the first “biological unit” as defined in the PDB. Calculations were performed on commodity cluster hardware running RedHat Enterprise Linux. Failed calculations were re-run up to five times, including at both Amgen and Schrödinger facilities, to ensure that failures were not the result of compute infrastructure issues. To identify protein-protein interaction interfaces, we checked whether any of the TSN molecules modeled into a predicted druggable site also overlapped with another protein chain in the crystal structure. Overlap was defined as at least one atom of the TSN molecule being within 2 Å of the additional protein chain, where hydrogens were included. To identify protein-ligand interfaces, we used the previously-described volume overlap calculation. Finally, to analyze the results of the mammalian proteins in the PDB for obligate dimers, we used the Interevol database, publicly available at http://biodev.cea.fr/interevol/interevol.aspx [31]. We downloaded the database (July 2012 release) and joined the data with our PDB results by matching both the four-letter PDB code and any chain identifier. PDB IDs were translated to gene ID's using the SWPROT database [36], and all gene annotations were performed using bioDBnet [37] Structure figures were produced using PyMOL version 1.4.1 [38].

To map MDDR drugs to PDB co-crystal structures, we first identified all oral drugs in MDDR that were annotated as ‘marketed’ and delivered orally as tablets or pills. PipelinePilot (ver. 8.5., Accelrys Software, San Diego, CA) was used to identify identical compounds based on structural identity when compared with the SMILES strings included in the HET code file downloaded from RCSB LigandDepot [30]. PDB codes were then identified that corresponded to matched HET codes.

Matlab version 7.9 (R2009b, The Mathworks Inc., Natick, MA) was used to generate Figures 2, 5, and 6, and also to calculate statistical means, variances, and two-sample Kolmogorov-Smirnov test results for the general validation set.

Calculations were performed on Intel Xeon CPU (2.7GHz) multi-core processors running RedHat Enterprise version 6. CPU timings quoted in the paper are per single core.

## Acknowledgments

We thank Nigel Walker, Philip Tagari, Yax Sun, Paul Kassner, Mike Ollman, and Astrid Ruefli-Brasse at Amgen, and Woody Sherman and Alessandro Monge at Schrödinger for their support and helpful discussions.

## Supporting Information

Table S1 **Protein-protein interaction targets from 2P2I that have less well-defined druggability as determined by conclusive results from multiple research groups, or where known drugs are metal chelators (HIV Integrase).** These targets have protein-protein co-crystal structures shown in the top half of the table, and corresponding protein-ligand co-crystal structures shown in the bottom half. Score is Dscore+, ‘cmpd’ indicates a small molecule compound, and volumes are in units of Å^3^. “*” indicates ‘not applicable’ because the site's initial Dscore+ values did not meet the cut-off for flexibility modeling. **^1^** Menin-MLL inhibitors has been reported in academic discovery efforts, but there does not appear to be enough evidence yet to definitively assign druggability. One group reports nM inhibitors with small molecule compounds (see Murai et al., *J. Biol Chem.* 2011 286: 31742–8), while another group reports nM inhibitors with large macrocyclic peptidomimetics that do not fall into drug-like property ranges (see Zhou et al., *J. Med Chem.* 2013 56: 1113–23). Several of the Menin-MLL inhibitor co-crystal structures contained several extra dummy atoms in the inhibitor binding site, and we removed them for the purposes of running our analysis. **^2^** HIV Integrase complex involves a DNA-protein interaction, and thus inhibitors are not protein-protein inhibitors. In addition, the approved drugs, raltegravir and elvitegravir, bind to two Mg2+ ions bound to HIV integrase, and thus are metal chelators (see Hare et al., *Nature*. 2010 464: 232–6). Metal chelators are not captured by structure-based druggability approaches, as discussed in the *Introduction*, although allosteric LEDGF/p75-Integrase inhibitors are showing promise.
(DOCX)

Table S2 **Potential cryptic druggable sites based on flexible modeling of the mammalian structural proteome.**
(DOCX)

Table S3 **Binding site residues for predictions in Table S2.** Notes: chains are appended to residue names with a leading period. In a few cases, more than one binding site may be defined.
(DOCX)

Table S4 **Extension of** **Table 2** **to comparison of druggability estimations on all targets from** ***Huang and Jacobson*** **** [17] **.** The data under the *Structural data* and *Docking-based druggability* are from reference 17. Aldose reductase sites required manual intervention to include NAP co-factor. Without co-factor, Dscore+ is lower, around 1.1. The DHFR structure with PDB ID 6dfr is missing a large portion of the binding site (both protein and co-factor), and so calculations would not be relevant; we indicated this with “[Not calculated]”. The flexible druggability method is only performed for binding sites that meet an initial score (with the rigid crystal structure). However, for the purposes of this study, we removed this cut-off in order to generate values for IL-2 and HPV E2. For IL-2, performing the flexibility modeling procedure results in DScore+ values of 1.5 (1z92), 1.5 (1py2), and 1.7 (1m48), with small, non-drug-like volumes of 98, 82, 53, respectively. For HPV E2, the DScore+ values are 1.1 (1tue) and 1.5 (1r6n), with reasonable drug-like volumes. For neuraminidase (NA), the Dscore+ values are 1.8 (1a4g), 1.7 (1a4q), and 1.7 (1nsc), with drug-like volumes.
(DOCX)
