# Correction: Structure-Based Druggability Assessment of the Mammalian Structural Proteome with Inclusion of Light Protein Flexibility

**DOI:** 10.1371/journal.pcbi.1003875

**Published:** 2014-09-09

**Authors:** The PLOS Computational Biology Staff

A number of figure legends are associated with the wrong figures in the published article.

The legend of Figure 6 should be associated with Figure 4.

The legend of Figure 4 should be associated with Figure 5.

The legend of Figure 5 should be associated with Figure 6.
