# Supplementary material for: Structure-Based Druggability Assessment of the Mammalian Structural Proteome with Inclusion of Light Protein Flexibility
Source: PLoS Comput Biol. 2014 Jul 31;10(7):e1003741. doi: 10.1371/journal.pcbi.1003741 (PMC4117425; doi:10.1371/journal.pcbi.1003741)
Supplement: Table S1 — Protein-protein interaction targets from 2P2I that have less well-defined druggability as determined by conclusive results from multiple research groups, or where known drugs are metal chelators (HIV Integrase). These targets have protein-protein co-crystal structures shown in the top half of the table, and corresponding protein-ligand co-crystal structures shown in the bottom half. Score is Dscore+, ‘cmpd’ indicates a small molecule compound, and volumes are in units of Å3. “*” indicates ‘not applicable’ because the site's initial Dscore+ values did not meet the cut-off for flexibility modeling. 1 Menin-MLL inhibitors has been reported in academic discovery efforts, but there does not appear to be enough evidence yet to definitively assign druggability. One group reports nM inhibitors with small molecule compounds (see Murai et al., J. Biol Chem. 2011 286: 31742–8), while another group reports nM inhibitors with large macrocyclic peptidomimetics that do not fall into drug-like property ranges (see Zhou et al., J. Med Chem. 2013 56: 1113–23). Several of the Menin-MLL inhibitor co-crystal structures contained several extra dummy atoms in the inhibitor binding site, and we removed them for the purposes of running our analysis. 2 HIV Integrase complex involves a DNA-protein interaction, and thus inhibitors are not protein-protein inhibitors. In addition, the approved drugs, raltegravir and elvitegravir, bind to two Mg2+ ions bound to HIV integrase, and thus are metal chelators (see Hare et al., Nature. 2010 464: 232–6). Metal chelators are not captured by structure-based druggability approaches, as discussed in the Introduction, although allosteric LEDGF/p75-Integrase inhibitors are showing promise. (DOCX) [file pcbi.1003741.s001.docx]

| **Target** | **Assigned** | **Small mol.** | **PDB** | **Crystal pocket** | | **Flexible model** | |
| --- | --- | --- | --- | --- | --- | --- | --- |
| **name** | **druggability** | **progression** | **ID** | Dscore+ | Volume | Dscore+ | Volume |
| XDM2 | Unknown | Discovery | 1ycq | 1.5 | 132 | 2.0 | 156 |
| HDM4 | Unknown | Discovery | 3dab | 1.4 | 125 | 1.8 | 145 |
| Menin ^1^ | Unknown | Early Disc. | 4gq6 | 1.3 | 210 | 1.3 | 184 |
| Integrase **^2^** | Difficult | Marketed **^2^** | 2b4j | 0.6 | 38 | * | * |
| HPV E2 | Difficult | Early Disc. | 1tue | 0.8 | 57 | * | * |
| XIAP | Difficult | Early Disc. | 1g73 | 0.8 | 59 | * | * |
| XIAP | Difficult | Early Disc. | 1nw9 | 1.2 | 311 | * | * |
| HDM4 | Unknown | Discovery | 3lbj | 1.5 | 117 | 2.3 | 174 |
| Menin ^1^ | Unknown | Early Disc. | 4gq4 | 1.3 | 200 | 1.5 | 177 |
| XIAP | Difficult | Early Disc. | 2jk7 | 0.7 | 65 | * | * |
| XIAP | Difficult | Early Disc. | 2opy | 0.8 | 71 | * | * |
| XIAP | Difficult | Early Disc. | 3clx | 0.9 | 80 | * | * |
| XIAP | Difficult | Early Disc. | 3cm2 | 0.8 | 68 | * | * |
| XIAP | Difficult | Early Disc. | 3eyl | 0.8 | 63 | * | * |
| XIAP | Difficult | Early Disc. | 3g76 | 0.8 | 73 | * | * |

**Table S1. Protein-protein interaction targets from 2P2I ^10^ that have less well-defined druggability as determined by conclusive results from multiple research groups, or where known drugs are metal chelators (HIV Integrase).** These targets have protein-protein co-crystal structures shown in the top half of the table, and corresponding protein-ligand co-crystal structures shown in the bottom half. Score is Dscore+, ‘cmpd’ indicates a small molecule compound, and volumes are in units of Å^3^. “*” indicates ‘not applicable’ because the site’s initial Dscore+ values did not meet the cut-off for flexibility modeling. **^1^** Menin-MLL inhibitors has been reported in academic discovery efforts, but there does not appear to be enough evidence yet to definitively assign druggability. One group reports nM inhibitors with small molecule compounds (see Murai et al., *J. Biol Chem.* 2011 286: 31742-8), while another group reports nM inhibitors with large macrocyclic peptidomimetics that do not fall into drug-like property ranges (see Zhou et al., *J. Med Chem.* 2013 56: 1113-23). **^2^** HIV Integrase complex involves a DNA-protein interaction, and thus inhibitors are not protein-protein inhibitors. In addition, the approved drugs, raltegravir and elvitegravir, bind to two Mg2+ ions bound to HIV integrase, and thus are metal chelators (see Hare et al., *Nature.* 2010 464: 232-6). Metal chelators are not captured by structure-based druggability approaches, as discussed in the *Introduction*, although allosteric LEDGF/p75-Integrase inhibitors are showing promise.
