# Supplementary material for: Structure-Based Druggability Assessment of the Mammalian Structural Proteome with Inclusion of Light Protein Flexibility
Source: PLoS Comput Biol. 2014 Jul 31;10(7):e1003741. doi: 10.1371/journal.pcbi.1003741 (PMC4117425; doi:10.1371/journal.pcbi.1003741)
Supplement: Table S2 — Potential cryptic druggable sites based on flexible modeling of the mammalian structural proteome. (DOCX) [file pcbi.1003741.s002.docx]

Table S2: Potential cryptic druggable sites based on flexible modeling of the mammalian structural proteome.

| **UniProt Entry** | **UniProt Protein** | **PDB ID** |
| --- | --- | --- |
| CALM_HUMAN | Calmodulin | 1cdl, 3bya, 4djc |
| GSTP1_HUMAN | Glutathione S-transferase P | 1eoh, 1kbn |
| GSTA1_MOUSE | Glutathione S-transferase A1 | 1f3b |
| GRAN_HUMAN | Grancalcin | 1f4q |
| DHB1_HUMAN | Estradiol 17-beta-dehydrogenase 1 | 1fds, 1yb1 |
| MBL1_RAT | Mannose-binding protein A | 1fih |
| CALM_RAT | Calmodulin | 1g4y, 2hqw, 2ygg |
| GSTP1_MOUSE | Glutathione S-transferase P 1 | 1glp, 1glq, 3o76 |
| TERF2_HUMAN | Telomeric repeat-binding factor 2 | 1h6p |
| MBL1_RAT | Mannose-binding protein A | 1kwv, 1kwx, 1kwy, 1kwz, 1kx0 |
| H2B11_XENLA | Histone H2B 1.1 | 1kx3, 1kx5, 1p3i, 1p3l |
| H32_XENLA | Histone H3.2 | 1kx5, 1p3i, 1p3l |
| GSTM1_RAT | Glutathione S-transferase Mu 1 | 1mtc, 2gst, 3gst, 4gst, 5gst, 5gst, 6gst, 6gsu, 6gsv, 6gsx |
| C1QBP_HUMAN | Complement component 1 Q subcomponent-binding protein | 1p32 |
| PPCS_HUMAN | Phosphopantothenate--cysteine ligase | 1p9o |
| GSTA1_HUMAN | Glutathione S-transferase A1 | 1pkz, 1xwg |
| HEM2_HUMAN | Delta-aminolevulinic acid dehydratase | 1pv8 |
| SFTPD_HUMAN | Pulmonary surfactant-associated protein D | 1pw9, 3ikp |
| GSK3B_HUMAN | Glycogen synthase kinase-3 beta | 1r0e |
| CYTC_HUMAN | Cystatin-C | 1r4c, 3qrd |
| CYH2_HUMAN | Cytohesin-2 | 1r8s |
| GBB1_BOVIN | Guanine nucleotide-binding protein G(I)/G(S)/G(T) subunit beta-1 | 1tbg |
| COX5A_BOVIN | Cytochrome c oxidase subunit 5A, mitochondrial | 1v55, 2dyr, 2dys |
| MYL6B_HUMAN | Myosin light chain 6B | 1w7j |
| PDE4D_HUMAN | cAMP-specific 3',5'-cyclic phosphodiesterase 4D | 1xon, 2pw3, 2qyn |
| GSTM1_HUMAN | Glutathione S-transferase Mu 1 | 1xw6 |
| FOXP2_HUMAN | Forkhead box protein P2 | 2a07 |
| FCN1_HUMAN | Ficolin-1 | 2d39 |
| PSF1_HUMAN | DNA replication complex GINS protein PSF1 | 2e9x |
| RD23B_MOUSE | UV excision repair protein RAD23 homolog B | 2f4m, 2f4o |
| CALM_BOVIN | Calmodulin | 2fot |
| GSTP1_PIG | Glutathione S-transferase P | 2gsr |
| ODB2_BOVIN | Lipoamide acyltransferase component of branched-chain alpha-keto acid dehydrogenase complex | 2ii3 |
| P5CR1_HUMAN | Pyrroline-5-carboxylate reductase 1 | 2izz |
| KIF5A_RAT | Kinesin heavy chain isoform 5A | 2kin |
| H3_DROME | Histone H3 | 2nqb |
| H2B_DROME | Histone H2B | 2nqb |
| SYN3_HUMAN | Synapsin-3 | 2p0a |
| HMCS1_HUMAN | Hydroxymethylglutaryl-CoA synthase, cytoplasmic | 2p8u |
| TNKS1_HUMAN | Tankyrase-1 | 2rf5 |
| ACTS_RABIT | Actin, alpha skeletal muscle | 2v52 |
| AAPK1_RAT | 5'-AMP-activated protein kinase catalytic subunit alpha-1 | 2v8q |
| PARVA_HUMAN | Alpha-parvin | 2vzi |
| WDR5_MOUSE | WD repeat-containing protein 5 | 2xl2 |
| LTOR2_MOUSE | Ragulator complex protein LAMTOR2 | 2zl1 |
| PDCD6_HUMAN | Programmed cell death protein 6 | 2zn9, 3aaj |
| RADI_MOUSE | Radixin | 2zpy |
| CAPZB_CHICK | F-actin-capping protein subunit beta isoforms 1 and 2 | 3aa6 |
| H2B1J_HUMAN | Histone H2B type 1-J | 3av1, 3av1, 3azg |
| H32_HUMAN | Histone H3.2 | 3av1 |
| CARM1_RAT | Histone-arginine methyltransferase CARM1 | 3b3g |
| B2CL1_HUMAN | Bcl-2-like protein 1 | 3fdl, 3fdm, 3r85 |
| CD1D1_MOUSE | Antigen-presenting glycoprotein CD1d1 | 3gmo |
| NAGAB_HUMAN | Alpha-N-acetylgalactosaminidase | 3h53 |
| COG4_HUMAN | Conserved oligomeric Golgi complex subunit 4 | 3hr0 |
| MCL1_MOUSE | Induced myeloid leukemia cell differentiation protein Mcl-1 homolog | 3io9 |
| MCL1_HUMAN | Induced myeloid leukemia cell differentiation protein Mcl-1 | 3kj0, 3kz0, 3mk8 |
| CAPZB_CHICK | F-actin-capping protein subunit beta isoforms 1 and 2 | 3lk4 |
| STAP1_HUMAN | Signal-transducing adaptor protein 1 | 3maz |
| CENPA_HUMAN | Histone H3-like centromeric protein A | 3nqu |
| SYMPK_HUMAN | Symplekin | 3o2q |
| PPARG_HUMAN | Peroxisome proliferator-activated receptor gamma | 3r8i |
| DLDH_HUMAN | Dihydrolipoyl dehydrogenase, mitochondrial | 3rnm |
| GLYG_HUMAN | Glycogenin-1 | 3u2v |
| KDM8_HUMAN | Lysine-specific demethylase 8 | 3uyj |
| S10A4_HUMAN | Protein S100-A4 | 3zwh |
| HDDC2_HUMAN | HD domain-containing protein 2 | 4dmb |
| CENPX_HUMAN | Centromere protein X | 4dra |
| CLOCK_MOUSE | Circadian locomoter output cycles protein kaput | 4f3l |
