# Supplementary material for: Structure-Based Druggability Assessment of the Mammalian Structural Proteome with Inclusion of Light Protein Flexibility
Source: PLoS Comput Biol. 2014 Jul 31;10(7):e1003741. doi: 10.1371/journal.pcbi.1003741 (PMC4117425; doi:10.1371/journal.pcbi.1003741)
Supplement: Table S3 — Binding site residues for predictions in Table S2. Notes: chains are appended to residue names with a leading period. In a few cases, more than one binding site may be defined. (DOCX) [file pcbi.1003741.s003.docx]

Table S3: Binding site residues for predictions in Table S2.

Notes: Chains are appended to residue names with a leading period. In a few cases, more than one binding site may be defined.

1cdl_A V55.A E31.A L112.A L116.A E114.A V91.A L18.A M109.A E84.A T146.A E140.A F68.A R74.A E11.A M144.A E47.A V142.A V136.A M36.A S101.A Q41.A K30.A E120.A T29.A I52.A L48.A T28.A I27.A G33.A M71.A L39.A M72.A R126.A D129.A F12.A A15.A E14.A V121.A A88.A I125.A F141.A I85.A L32.A Q49.A F89.A L105.A D20.A S17.A V35.A N137.A E87.A E127.A M145.A I63.A M51.A E123.A A128.A F16.A I100.A N53.A V108.A E54.A F92.A F19.A M124.A

1eoh_B F8.B V10.B E130.B N93.B Y108.B F129.B P9.B P53.B V92.B V144.B T131.B G124.B P128.B L126.B A87.B D90.B R11.B G205.B V96.B V89.B L132.B I203.B V35.B K102.B I104.B L88.B I107.B R13.B Q51.B Y7.B L155.B T34.B D94.B V33.B P202.B Q84.B N206.B G12.B K127.B L158.B N204.B Q135.B Q125.B L99.B G95.B L52.B I143.B M91.B W38.B D98.B

1f3b_A I212.A L110.A V155.A D208.A L180.A F135.A Y131.A I98.A L139.A L71.A R216.A A134.A L101.A I105.A D100.A R68.A H142.A T127.A L90.A P206.A L132.A E136.A R12.A G13.A G106.A T67.A E161.A L163.A E16.A R203.A R130.A V148.A L147.A Y94.A Y165.A E103.A Q107.A L174.A V109.A S95.A L99.A P205.A T102.A E168.A I18.A W20.A V138.A A11.A G97.A V162.A C17.A K137.A M15.A M93.A R14.A E96.A L159.A M207.A V166.A

1f4q_A D204.A R154.A T189.A F210.A G213.A K194.A A180.A T162.A Q212.A Q200.A F191.A T159.A G152.A Q199.A I163.A R186.A L185.A D196.A Y207.A R195.A G201.A L188.A I217.A L155.A D190.A A187.A R166.A F192.A T214.A L211.A C181.A K184.A K193.A I148.A Y153.A Y167.A A203.A F205.A I206.A S202.A D209.A

1fih_B V113.B I147.B H116.B M119.B C128.B F112.B V124.B F111.B G104.B K110.B E117.B C222.B A151.B A220.B A100.B K152.B E223.B L127.B L131.B F224.B M103.B V150.B E149.B E142.B K145.B F101.B N96.B S102.B H99.B K105.B N115.B K106.B T134.B R132.B G133.B V221.B T114.B A146.B

1g4y_R V136.R I85.R F141.R L105.R T110.R I100.R M124.R Y99.R A128.R G113.R K115.R F89.R A88.R N137.R F92.R L112.R V142.R M145.R D93.R R106.R E104.R S101.R E114.R V108.R E139.R Y138.R I125.R L116.R M109.R K94.R R90.R V91.R

1glp_A P53.A G12.A Q135.A I203.A V10.A T131.A L170.A F129.A W38.A N93.A Y108.A I107.A G207.A N204.A H125.A P128.A V92.A L158.A T105.A L132.A L176.A N206.A P202.A M89.A I35.A K102.A V104.A L52.A T34.A H162.A G124.A Q51.A L155.A Q88.A M39.A L133.A Y103.A L99.A I161.A L122.A A151.A R11.A D98.A M91.A P9.A G50.A E130.A R13.A V33.A N154.A G95.A I143.A Y7.A R100.A D36.A G101.A V144.A F8.A V96.A L159.A L126.A K127.A G205.A T109.A D94.A K44.A

1glq_A P53.A G12.A Q135.A I203.A V10.A Q163.A L170.A F129.A W38.A N93.A Y108.A I107.A N204.A H125.A P128.A V92.A L158.A G168.A T105.A L132.A L176.A N206.A P202.A M89.A I35.A K102.A V104.A P123.A L52.A T34.A H162.A Y118.A G124.A Q51.A P167.A L155.A Q88.A M39.A Y103.A L99.A C14.A D90.A I161.A L122.A R11.A V164.A D98.A A121.A A87.A M91.A L165.A P9.A G50.A L160.A E130.A R13.A V33.A A166.A G95.A R18.A I143.A Y7.A R100.A C169.A D36.A G101.A V144.A F8.A V96.A L159.A L126.A V32.A V180.A K127.A G205.A T109.A D94.A K44.A

1h6p_B A1085.B L1091.B T1096.B H1095.B I1079.B R1080.B M1083.B E1112.B C1106.B K1058.B F1059.B V1088.B S1098.B M1104.B D1081.B V1097.B F1061.B G1092.B L1100.B Q1084.B Y1060.B L1057.B L1087.B L1107.B P1090.B Q1105.B K1093.B R1077.B F1076.B R1102.B R1089.B E1094.B R1109.B S1108.B L1101.B A1064.B R1099.B R1054.B

1kwv_A F112.A M119.A N115.A C217.A A215.A A100.A K110.A F101.A F111.A L127.A G104.A T134.A M103.A S102.A T114.A E218.A F219.A L131.A N96.A K97.A V113.A H99.A R132.A E130.A E117.A L98.A T95.A G133.A V216.A C128.A P220.A V124.A H116.A K105.A

1kwv_C G133.C V216.C E218.C K97.C P220.C K105.C F112.C N96.C H99.C L127.C C128.C C217.C R132.C T114.C A100.C A215.C N115.C F111.C T134.C F219.C T95.C E130.C V113.C L131.C M103.C E93.C

1kwx_C G108.C K106.C A221.C V216.C E218.C K97.C P220.C F101.C K105.C S90.C G104.C F112.C N96.C E117.C M119.C H99.C L127.C C128.C V124.C C217.C K109.C R132.C S107.C T114.C L98.C A100.C A215.C N115.C F111.C K110.C H116.C F219.C T95.C E130.C V113.C S102.C L131.C M103.C L94.C E93.C

1kwy_B V113.B I147.B H116.B M119.B P220.B E130.B C128.B F112.B F111.B G104.B K110.B E143.B E117.B A151.B K97.B A100.B N140.B K152.B L127.B A215.B L131.B K109.B M103.B C217.B V150.B E149.B E142.B E93.B K145.B F101.B N96.B S102.B H99.B K105.B N115.B K106.B T134.B R132.B V216.B G133.B A221.B T114.B F219.B E218.B A146.B L98.B

1kwy_C G108.C K106.C G133.C E143.C V135.C V216.C K97.C E218.C A136.C R139.C P220.C F101.C K105.C G104.C F112.C N96.C E117.C M119.C K152.C H99.C L127.C C128.C V124.C C217.C I137.C R132.C N140.C K109.C T114.C A100.C A215.C N115.C L92.C F111.C E149.C A146.C K110.C I147.C K145.C V150.C H116.C T134.C A141.C T95.C F219.C E130.C V113.C S102.C E142.C L131.C S129.C M103.C

1kwz_A N144.A F112.A K109.A N115.A C217.A I147.A A100.A K110.A F101.A F111.A L127.A N140.A K106.A G104.A A141.A M103.A S102.A V150.A L92.A T114.A E218.A F219.A L131.A E143.A K152.A N96.A K145.A K97.A H99.A V113.A A151.A R132.A E130.A T95.A G133.A V216.A P220.A E149.A A146.A K105.A E93.A E142.A

1kx0_A F112.A M119.A K109.A N115.A C217.A I147.A A215.A A100.A K110.A F111.A L127.A N140.A K106.A G104.A A141.A M103.A S102.A V150.A T114.A E218.A F219.A L131.A E143.A K152.A N96.A K145.A K97.A H99.A V113.A R132.A E130.A T95.A G133.A C128.A H116.A E149.A A146.A K105.A E93.A E142.A

1kx0_C G108.C K106.C G133.C E143.C A221.C E218.C K97.C P220.C F101.C K105.C A151.C G104.C F112.C N96.C K152.C H99.C L127.C C128.C C217.C R132.C K109.C S107.C T114.C L98.C A100.C A215.C N115.C F111.C E149.C A146.C K110.C I147.C V150.C K145.C H116.C T134.C F219.C T95.C E130.C V113.C E142.C L131.C M103.C E93.C

1kx3_D I86.D S88.D A74.D E68.D N64.D L99.D I91.D F67.D V95.D R89.D L98.D A114.D G111.D Q92.D V108.D M59.D A107.D Y34.D L103.D E110.D H106.D V63.D T93.D F62.D A71.D V66.D S109.D I70.D V115.D T112.D

1kx3_H V45.H I36.H H106.H T116.H F62.H L103.H L42.H G50.H S88.H V95.H H46.H V41.H Y34.H E110.H V108.H V115.H A55.H K40.H A35.H I58.H T49.H I86.H I91.H V66.H V63.H A71.H F67.H A114.H N60.H A107.H S52.H A104.H R69.H G111.H L99.H I70.H I51.H R89.H K54.H T112.H L98.H S109.H E68.H Y39.H V38.H Y118.H T87.H Y37.H M59.H M56.H Q92.H

1kx5_A S96.A K56.A E50.A F104.A I119.A R63.A T107.A N108.A S57.A Q93.A L61.A I62.A L48.A A102.A R53.A L103.A T58.A L100.A R52.A E105.A V101.A Q55.A A47.A L60.A I51.A F67.A E94.A E97.A A98.A E59.A Y54.A

1kx5_E E97.E Q93.E E105.E A47.E K56.E L61.E A98.E N108.E L100.E T58.E V101.E Q55.E F104.E S57.E F67.E L48.E I51.E A102.E S96.E L92.E R52.E I62.E E59.E L60.E Y54.E E94.E I119.E V46.E E50.E

1kx5_H H106.H T116.H F62.H L103.H T93.H R96.H S88.H V95.H A74.H Y34.H E110.H V108.H N64.H V115.H I86.H I91.H V66.H A71.H V63.H F67.H T119.H A107.H A104.H G111.H L99.H I70.H R89.H T112.H L98.H S109.H E68.H Y118.H T87.H Q92.H

1mtc_A S138.A V155.A Y166.A L180.A E132.A L136.A V99.A Y137.A I98.A Q102.A L158.A V103.A L163.A L141.A R107.A K133.A D161.A R95.A E139.A Q109.A M134.A I94.A I162.A M104.A A96.A Y160.A N106.A A148.A A159.A F140.A F147.A

1p32_B E127.B L182.B L184.B D177.B A181.B I173.B V170.B L235.B Q212.B G178.B S210.B Y224.B R246.B V172.B M240.B L239.B L231.B K179.B V209.B K174.B F211.B I206.B L243.B L226.B K180.B F242.B H238.B D176.B N175.B

1p3i_A E497.A E459.A A475.A R453.A V489.A I462.A I474.A Q455.A K456.A A498.A A488.A L460.A F484.A A491.A T507.A E450.A L503.A L470.A F467.A Y454.A L482.A L448.A K479.A I524.A F504.A S457.A L461.A A447.A I451.A E494.A Q485.A T458.A P466.A V471.A E505.A F478.A L500.A L492.A I519.A Q493.A Q476.A S496.A Y499.A V501.A Q468.A R472.A

1p3i_D Y1318.D N1264.D F1262.D Q1292.D A1271.D A1307.D T1312.D K1313.D E1310.D A1314.D V1308.D V1266.D S1288.D T1287.D I1291.D V1315.D I1270.D R1289.D A1304.D Y1234.D L1298.D G1311.D V1263.D L1299.D F1267.D H1306.D S1309.D V1295.D

1p3i_H T1449.H M1459.H V1495.H A1435.H L1503.H N1464.H A1507.H I1491.H E1510.H A1471.H I1451.H I1470.H E1468.H L1442.H L1499.H V1445.H E1502.H V1466.H I1458.H T1512.H M1456.H V1508.H G1450.H V1438.H Q1492.H N1460.H A1474.H S1457.H S1433.H V1463.H A1455.H Y1439.H A1504.H I1486.H G1511.H H1506.H F1462.H V1441.H S1488.H Y1434.H Y1437.H H1446.H F1467.H T1493.H

1p3l_A E497.A E459.A R453.A I462.A Q455.A A502.A K456.A A498.A L460.A T507.A E450.A L503.A H518.A L470.A F467.A R528.A Y454.A L448.A I524.A F504.A S457.A L461.A R452.A A447.A V517.A N508.A I451.A E494.A T458.A E505.A L500.A L492.A I519.A Q493.A S496.A Y499.A V501.A

1p3l_D N1264.D F1262.D M1259.D Q1292.D M1256.D V1238.D T1249.D E1302.D A1271.D A1307.D L1242.D T1312.D T1293.D H1246.D L1303.D E1310.D A1274.D V1308.D V1266.D S1288.D Y1237.D V1241.D I1258.D E1268.D A1235.D T1287.D I1251.D I1291.D V1315.D I1270.D R1289.D N1260.D A1304.D I1286.D Y1234.D L1298.D G1311.D V1263.D L1299.D V1245.D Y1239.D A1255.D S1252.D F1267.D H1306.D S1309.D V1295.D

1p3l_E E659.E E697.E F704.E S657.E I674.E Q693.E L661.E Y654.E I719.E R652.E S696.E A698.E N708.E E694.E L660.E T658.E L670.E A695.E A702.E P666.E K656.E V701.E E705.E I662.E L700.E E650.E Q655.E F667.E I651.E

1p9o_B P11.B P48.B R55.B P211.B E130.B M191.B G127.B G288.B P53.B E137.B E290.B A131.B A75.B P187.B V188.B A93.B A153.B T150.B T143.B L74.B L132.B L214.B V186.B S103.B F96.B Y135.B Q12.B F184.B A70.B L57.B V7.B V210.B P97.B L102.B Y92.B F128.B L105.B W101.B P98.B Q136.B L152.B E9.B P91.B Y185.B K287.B L49.B F56.B V47.B D154.B H94.B R52.B V146.B T68.B R133.B V289.B R106.B S134.B A138.B Q99.B A72.B E50.B V54.B E293.B L125.B R95.B F90.B E71.B F10.B T151.B K46.B L118.B A129.B F144.B A76.B A51.B G77.B P126.B

1pkz_B R131.B R217.B V55.B P206.B K138.B E169.B G98.B P134.B I219.B P207.B Y9.B E137.B H143.B I128.B L107.B D101.B V167.B G14.B E162.B A216.B M208.B R155.B P56.B D209.B E215.B G103.B Q54.B R69.B E104.B R45.B Y166.B A100.B L160.B S18.B E97.B L41.B F136.B L163.B E17.B I99.B R13.B V139.B L102.B L181.B N11.B M94.B I96.B R15.B E214.B F133.B V149.B Y132.B T68.B K127.B M16.B L213.B L148.B L22.B V111.B I106.B A135.B Y95.B L108.B A12.B F10.B I35.B L164.B P110.B H159.B L72.B L109.B S212.B L140.B

1pv8_B A118.B C75.B R74.B N30.B D120.B V164.B I316.B A274.B Q163.B L77.B C162.B V121.B Y276.B V117.B L249.B I315.B M250.B L273.B I78.B D247.B I314.B V76.B D313.B A28.B Y196.B A166.B F79.B I32.B P272.B L31.B V275.B M248.B L116.B C119.B V165.B M194.B S29.B

1pw9_C Y228.C C353.C E354.C K246.C S226.C F225.C L233.C G241.C Q267.C A264.C K230.C V352.C S239.C C261.C V231.C L260.C V351.C A248.C E242.C G265.C F245.C Q222.C K229.C F355.C N236.C L221.C A257.C T247.C G266.C E232.C G249.C I244.C

1r0e_A L112.A F229.A I84.A L198.A Y288.A P225.A Q185.A I270.A W241.A V70.A F201.A L266.A L132.A D200.A T289.A P136.A G202.A F293.A L273.A I62.A V82.A Y134.A C199.A N64.A L188.A L130.A V263.A G63.A E137.A D133.A V135.A I109.A P294.A I269.A Y71.A I296.A P276.A T275.A K292.A K85.A A83.A D264.A R141.A V267.A M284.A Q265.A F291.A F67.A Y323.A N186.A T138.A M101.A K183.A R111.A G274.A I228.A Q72.A I281.A V110.A E97.A E268.A

1r4c_B V23.B S17.B E19.B K54.B Y34.B A16.B Q48.B E33.B R45.B G22.B R24.B V49.B A46.B V31.B S44.B Q55.B D28.B A52.B F29.B A26.B A30.B N35.B R51.B A37.B L47.B L27.B R53.B V18.B E20.B

1r8s_E L195.E P154.E K156.E R162.E P246.E L153.E V204.E Y186.E F151.E I245.E E165.E A157.E S191.E Y190.E Q158.E D161.E W149.E M208.E I193.E F243.E R152.E I160.E H200.E K159.E N201.E M194.E D198.E M164.E L148.E N196.E T197.E S189.E R212.E K244.E G155.E L144.E

1tbg_C S207.C T221.C K15.C I338.C S281.C L51.C L300.C D20.C I37.C T86.C D254.C Q259.C A299.C S67.C T29.C K23.C L14.C L261.C V276.C H225.C S84.C F222.C L284.C R251.C T263.C I18.C L70.C L69.C R283.C C250.C F253.C M325.C L255.C Q220.C K209.C P236.C W339.C A28.C D27.C A26.C I33.C A326.C Y264.C R19.C Y85.C E260.C F235.C G224.C G282.C W63.C R49.C D66.C N340.C T34.C L285.C G324.C D323.C N16.C L30.C R22.C N239.C T223.C A242.C R68.C S279.C R256.C S277.C T321.C A257.C A24.C F234.C L252.C L286.C C233.C D258.C C25.C A11.C V327.C T87.C F241.C M262.C F278.C R48.C A240.C

1v55_R A54.R N59.R R56.R D60.R R53.R L96.R F61.R L29.R T35.R E69.R V42.R L36.R L93.R S63.R D73.R L106.R L58.R S99.R V65.R G97.R A26.R P43.R I48.R L68.R A62.R V70.R A51.R R30.R N34.R V37.R I67.R C55.R A64.R L104.R R57.R K31.R V71.R F19.R L52.R G38.R T92.R I98.R K74.R R66.R M33.R A75.R

1w7j_B T44.B R94.B L113.B L145.B Q31.B G92.B V95.B G53.B T127.B E126.B E118.B I148.B R110.B D34.B G33.B E47.B M120.B F96.B V48.B V128.B N45.B A46.B R63.B P55.B F93.B K119.B N42.B L60.B N54.B Y141.B L49.B L129.B C32.B Y89.B G106.B K56.B V51.B Y29.B K61.B S57.B H132.B M36.B I139.B V112.B L90.B S30.B L52.B E124.B G117.B T114.B D88.B E59.B V125.B V104.B H147.B F144.B L109.B R37.B L149.B A130.B

1xon V264.A S274.A S320.A I248.A T271.A ZN1001.A M357.A P322.A H200.A A196.A Q256.A L399.A V268.A M337.A F249.A V365.A H315.A R261.A P356.A E339.A A192.A V377.A I376.A N209.A L146.A H164.A Y329.A Y159.A I194.A F340.A T333.A D247.A L188.A D201.A A198.A L149.A L191.A N321.A I336.A M122.A L234.A S368.A L319.A G237.A D272.A V207.A K254.A F372.A R257.A L252.A V168.A Q343.A I265.A M273.A G206.A C246.A L241.A F238.A Q242.A I373.A Q369.A W332.A D318.A L142.A K239.A H160.A S208.A Y145.A L260.A H204.A Q210.A L240.A I199.A F195.A L229.A Q258.A

1xw6_A S138.A H107.A L180.A E132.A H166.A L136.A Y137.A I98.A Q102.A L158.A L163.A T103.A L141.A K133.A R95.A L130.A N101.A E139.A Q109.A L99.A L134.A M104.A V162.A D105.A Y160.A N106.A V159.A F140.A F147.A

1xw6_B V159.B E100.B G11.B M112.B E132.B M108.B G111.B H14.B M104.B L141.B E139.B Q102.B F147.B L59.B V207.B V162.B S138.B Y137.B Y115.B L165.B L163.B L110.B L180.B R10.B H166.B L136.B H107.B L76.B F208.B Q109.B Y126.B S209.B N73.B Y6.B N106.B V155.B D161.B P60.B L158.B I9.B I98.B T70.B K135.B L99.B F157.B A15.B A13.B I16.B F140.B K133.B N101.B L134.B Q71.B R95.B L130.B L12.B N58.B T103.B D105.B S72.B

1xwg_B R131.B R217.B V55.B P206.B K205.B K138.B E169.B G98.B I219.B P207.B Y9.B L184.B E137.B I158.B I128.B L107.B E68.B V167.B D101.B G14.B E162.B A216.B M208.B P56.B D209.B E215.B Q54.B G103.B E104.B Y166.B F220.B A100.B F222.B L160.B S18.B E97.B L41.B V161.B A156.B F136.B L163.B E17.B I99.B R13.B V139.B L102.B Y165.B L181.B L44.B N11.B D157.B M94.B I96.B R15.B F133.B V149.B Y132.B M16.B L213.B L148.B V111.B I106.B A135.B Y95.B L170.B L108.B S142.B A12.B F10.B I35.B L91.B L164.B P110.B L109.B H159.B L72.B S212.B L140.B

1yb1_A F144.A L198.A L194.A I203.A T195.A N115.A V206.A T37.A A153.A V87.A D88.A Q128.A T147.A K134.A A149.A T127.A Y120.A H143.A F190.A A142.A T193.A V118.A P130.A L201.A K148.A H41.A D62.A L151.A N64.A H191.A A116.A S122.A Q131.A N91.A F176.A W61.A T208.A V138.A I132.A A126.A I162.A C89.A P152.A S90.A T155.A G117.A E197.A I63.A T135.A K156.A T121.A G189.A E133.A V119.A G38.A

2a07_H E519.H A516.H T537.H I517.H Y540.H Y531.H F534.H I530.H L512.H T508.H T511.H F541.H F507.H Y509.H P506.H I513.H W533.H T535.H R536.H N544.H Q515.H F538.H

2d39_A W131.A L183.A Q159.A L145.A G178.A R170.A Q177.A L196.A F179.A T143.A L188.A Q182.A N192.A V157.A E185.A M321.A G180.A W187.A A174.A H132.A R140.A F186.A F158.A R160.A P141.A V144.A L142.A G189.A S181.A

2dyr_E S99.E R57.E L41.E R30.E R53.E P43.E A54.E I48.E V42.E V65.E C55.E I67.E F19.E A64.E D60.E Y39.E L93.E A75.E T35.E S63.E I98.E I47.E A51.E G97.E F61.E R66.E G38.E L96.E V37.E N34.E T92.E N59.E L29.E L52.E L36.E K74.E M33.E V71.E A62.E R56.E L68.E D40.E V70.E A26.E

2dys_E S99.E L41.E P43.E C55.E I67.E E69.E D60.E T35.E S63.E I47.E A51.E G97.E R66.E L96.E T92.E N59.E L29.E L52.E K74.E V71.E R56.E D40.E A26.E R30.E R53.E I48.E V42.E V65.E F19.E A64.E Y39.E L58.E L93.E L104.E A75.E I98.E F61.E G38.E V37.E N34.E L36.E K31.E M33.E A62.E D73.E L68.E V70.E

2e9x_C G1118.C L1175.C A1012.C S1010.C L1013.C M1171.C L1139.C S1135.C I1149.C T1141.C S1121.C L1124.C E1172.C F1142.C G1014.C Y1117.C T1178.C F1146.C D1169.C G1174.C I1134.C F1116.C I1143.C L1109.C R1145.C L1138.C G1120.C

2f4m_B Q305.B Q287.B A301.B Q284.B Q294.B L299.B N295.B I291.B I306.B L303.B E309.B R289.B L313.B Q304.B L298.B F285.B I317.B F324.B R308.B L279.B L328.B M288.B L302.B I292.B P283.B

2f4o_B Q305.B Q287.B A301.B E277.B L314.B Q284.B Q294.B L299.B H320.B I291.B I306.B L303.B E309.B L313.B R280.B H274.B Q304.B L298.B Q316.B F285.B M327.B I317.B F324.B R308.B Q290.B L279.B L328.B Q282.B L276.B M288.B L302.B N310.B I292.B P283.B H323.B

2fot_A V55.A L112.A E114.A V91.A L18.A G113.A E140.A F68.A M144.A T26.A K21.A M36.A Q41.A S81.A T29.A I52.A L48.A T28.A G33.A L39.A R126.A F12.A E14.A A88.A L69.A F141.A I125.A L32.A F89.A S17.A V35.A G61.A E127.A M51.A A128.A N53.A I100.A V108.A M124.A D64.A S38.A M109.A E84.A T146.A N111.A E104.A A147.A E83.A V142.A V136.A S101.A E67.A T34.A T110.A G40.A I27.A M71.A M72.A A15.A F65.A I85.A L105.A D20.A P43.A E87.A M145.A I63.A F16.A E54.A F92.A D56.A F19.A

2gsr_B M35.B Q62.B V94.B P9.B L120.B P200.B L153.B R11.B D150.B E95.B I159.B P51.B L174.B Y7.B D92.B D155.B E128.B T34.B H123.B V33.B L156.B K125.B A102.B F127.B L104.B L97.B N202.B W38.B V90.B P126.B R98.B T103.B Q49.B F8.B L168.B V10.B N152.B Y101.B A149.B T107.B L157.B I201.B V141.B M89.B I105.B K100.B D96.B E122.B L124.B R13.B L154.B N91.B G203.B C99.B L131.B S63.B L50.B Y106.B G12.B N64.B N204.B V142.B G93.B R158.B R48.B L130.B H160.B

2gst_B P206.B G11.B A33.B V103.B A159.B M104.B L141.B W7.B Y32.B F147.B A96.B L59.B K49.B M134.B Y166.B N216.B Y115.B R107.B L163.B L110.B Y40.B L136.B Q109.B F208.B V9.B S209.B K210.B N106.B V155.B D161.B Q165.B M34.B L158.B F140.B K133.B R95.B R144.B S72.B I207.B I111.B E132.B M108.B I162.B Q102.B G35.B S138.B Y137.B L180.B R17.B R10.B V99.B W146.B L211.B I94.B F183.B Y6.B T13.B Y160.B P60.B I98.B N8.B Q71.B A148.B W45.B L12.B R42.B N58.B D105.B

2hqw_A H107.A V55.A L112.A L116.A E114.A V91.A L18.A F68.A R74.A E11.A M144.A M36.A Q41.A I52.A T28.A A102.A G33.A L39.A R126.A E14.A V121.A A88.A F141.A I125.A L32.A F89.A V35.A N137.A E127.A M51.A E123.A A128.A N53.A I100.A V108.A M124.A E31.A M109.A E104.A V136.A S101.A T110.A I27.A M71.A M72.A A15.A L105.A R106.A M145.A I63.A F16.A E54.A F92.A D56.A F19.A

2ii3_D E411.D K421.D L293.D A310.D T311.D E225.D L226.D I306.D I352.D A286.D F246.D A414.D N412.D A361.D L337.D P350.D K349.D I359.D L233.D K243.D F415.D K234.D M287.D V351.D A237.D M416.D E308.D P413.D I340.D S305.D L353.D I236.D G345.D V295.D L420.D A348.D Y409.D N339.D V227.D I343.D M202.D D288.D R230.D T205.D I294.D R240.D T336.D M206.D L244.D G341.D E232.D F249.D I242.D S338.D S245.D I309.D L417.D P248.D G344.D S342.D G360.D L410.D L229.D F238.D F307.D K228.D L418.D

2ii3_G G360.G A237.G P355.G L410.G R230.G K243.G A208.G L233.G A310.G I285.G Y409.G F246.G T311.G V351.G M416.G T205.G I242.G S245.G M206.G F238.G A286.G L330.G N297.G L256.G R240.G N412.G L420.G N339.G I236.G E411.G S305.G A414.G L226.G V295.G K204.G I359.G K234.G L229.G F250.G T336.G V227.G F307.G I340.G S338.G I306.G L417.G L353.G G333.G P248.G L293.G F198.G I352.G M202.G I294.G L337.G L418.G F249.G K421.G K252.G A253.G P354.G E356.G A201.G L244.G L223.G K349.G F415.G P413.G P350.G I343.G M287.G E308.G A209.G S207.G E225.G D288.G A361.G

2ii3_H N282.H A414.H M206.H L417.H R230.H L362.H K234.H M287.H G333.H G292.H L244.H L325.H L229.H S321.H I309.H F249.H S305.H K200.H M202.H L316.H N297.H I340.H F335.H L418.H V295.H I242.H L293.H L251.H L353.H P413.H F198.H I343.H E356.H K349.H P248.H L313.H S326.H A201.H I352.H P350.H D288.H M247.H V227.H G341.H Q291.H I365.H I236.H A237.H F307.H G323.H G332.H V351.H L337.H N314.H L226.H P355.H G320.H E312.H L410.H V298.H P354.H E308.H A310.H V203.H L233.H F246.H I283.H T289.H F415.H T334.H T205.H Q317.H P296.H E225.H R240.H T336.H I331.H G284.H K243.H G360.H L420.H S245.H H199.H S342.H A286.H Y409.H T311.H I285.H N412.H R315.H I294.H K318.H N339.H M416.H E290.H F238.H T327.H L330.H S338.H P235.H I306.H I359.H F250.H

2izz_B V203.B D229.B G225.B I75.B L201.B M271.B V231.B D168.B A207.B M216.B I239.B A241.B L185.B V193.B F250.B E246.B A202.B A214.B G192.B L217.B L242.B N230.B P72.B T171.B S232.B A237.B L268.B G212.B R251.B A209.B A187.B D273.B I74.B A272.B H73.B G98.B K228.B T265.B A181.B A97.B A189.B H223.B L245.B I101.B G206.B M195.B G191.B T238.B S103.B L205.B K107.B I104.B L210.B L188.B S94.B L254.B D186.B P224.B V99.B F77.B L211.B R264.B Q269.B A213.B T100.B R266.B A184.B L197.B P76.B L227.B V244.B S270.B L79.B

2kin_B L288.B K328.B S258.B T298.B Q322.B S291.B L262.B A324.B V277.B E313.B L269.B L292.B G263.B F320.B N329.B V301.B R323.B N254.B K325.B C296.B N264.B M319.B R297.B I300.B L259.B A261.B T285.B V265.B G321.B T326.B I266.B I327.B I287.B M284.B S260.B S316.B T317.B D293.B T299.B I302.B

2nqb_A E459.A Q455.A K456.A A498.A L460.A E450.A L503.A Y454.A I524.A L461.A N508.A I451.A T458.A E505.A S496.A E497.A R453.A I462.A T507.A L470.A F467.A F504.A S457.A L500.A I519.A Q493.A V501.A

2nqb_E E659.E S657.E Q693.E L661.E G702.E L703.E S696.E L660.E T658.E L670.E T718.E I662.E L700.E I712.E E650.E Q655.E I651.E E697.E F704.E Y654.E I719.E A698.E N708.E T707.E K656.E V701.E I724.E E705.E R653.E V717.E F667.E

2nqb_H T1487.H L1503.H A1507.H I1491.H I1470.H K1513.H R1489.H R1496.H V1515.H V1508.H R1469.H A1514.H I1486.H H1506.H S1488.H F1467.H V1495.H E1510.H S1509.H Y1518.H L1499.H T1512.H Q1492.H I1466.H A1504.H G1511.H F1462.H T1519.H T1493.H

2p0a_A E227.A F231.A Q228.A K204.A T229.A P205.A A281.A L211.A S203.A L225.A I212.A E284.A V226.A H216.A T282.A W206.A Y280.A H251.A F215.A F230.A P232.A S209.A F208.A G250.A L249.A K248.A

2p8u_B Q223.B K219.B L51.B I222.B F319.B V162.B F311.B L270.B G166.B E316.B N167.B S274.B D312.B L306.B SCY129.B T171.B K317.B L275.B V315.B D314.B V216.B V304.B L229.B A168.B Q272.B K46.B H264.B Y345.B M320.B C224.B N343.B F204.B R169.B A164.B L280.B R313.B L220.B T309.B K269.B Y375.B Y225.B P266.B G218.B I215.B M278.B P214.B Y163.B R277.B Y267.B T165.B V271.B S221.B D217.B K273.B D203.B L226.B G50.B Y213.B S377.B Y310.B A276.B

2pw3_A A235.A Y223.A S320.A I248.A M337.A F249.A N201.A E339.A V377.A K262.A Y375.A Y329.A E230.A Y159.A T333.A S355.A D247.A H233.A E243.A L234.A L319.A R257.A L252.A M273.A V236.A H232.A L241.A Q242.A I373.A ZN501.A Q369.A D318.A K239.A H160.A Y145.A L260.A Q210.A L240.A I199.A F195.A V264.A T271.A M357.A P322.A A196.A Q256.A E347.A V268.A R261.A P356.A A192.A I376.A N209.A Q250.A H164.A F340.A R335.A C358.A N321.A L269.A I336.A S368.A G237.A V207.A F372.A N231.A Q343.A I265.A G206.A F238.A W332.A S208.A H204.A L229.A G371.A Q258.A

2qyn_A A235.A S320.A I248.A F135.A R346.A F126.A F249.A E339.A V377.A K262.A Y329.A Y159.A T333.A S355.A D247.A L188.A T253.A L149.A L131.A E243.A M122.A L234.A L319.A H276.A R257.A L252.A M273.A C246.A L241.A Q242.A I373.A ZN501.A Q369.A D318.A K239.A L142.A H160.A Y145.A L260.A Q210.A I199.A F195.A V264.A S274.A I137.A M357.A P322.A A196.A Q256.A H315.A R261.A P356.A A192.A N209.A I376.A L146.A Q250.A H164.A F340.A C358.A L191.A N321.A I336.A G237.A M277.A V207.A F372.A Q343.A I265.A G206.A F238.A W332.A S259.A S208.A H204.A L229.A G371.A Q258.A

2rf5 Q1248.A Y1213.A Y1203.A G1227.A I1228.A K1267.A A1210.A S1186.A G1185.A K1238.A S1221.A A1215.A I1189.A F1183.A T1232.A E1199.A Y1226.A T1307.A F1197.A G1206.A M1249.A D1198.A G1205.A R1247.A V1293.A H1246.A K1220.A G1233.A F1214.A P1187.A Y1224.A C1234.A A1202.A G1231.A G1211.A N1190.A G1209.A I1204.A H1184.A G1196.A R1149.A V1225.A E1291.A H1201.A I1192.A I1212.A F1188.A G1230.A P1235.A

2v52_B Y143.B G168.B C374.B L346.B A144.B E167.B G342.B Y169.B T148.B V134.B L104.B F352.B A347.B F375.B T351.B T106.B R147.B G146.B I136.B I345.B K373.B V139.B M355.B Y133.B L349.B A135.B G343.B Q354.B L140.B

2v8q_A Y452.A L432.A E469.A R464.A A547.A R433.A L460.A L546.A M538.A R435.A R401.A S402.A L451.A I543.A V427.A N541.A L461.A I400.A Q548.A E423.A A540.A F463.A D467.A Q450.A T458.A Y459.A G399.A S448.A M410.A K446.A V413.A I409.A D408.A H397.A E412.A R405.A M447.A I466.A Q403.A F535.A Y431.A W424.A L449.A C539.A S404.A V434.A L542.A F536.A V426.A D462.A K425.A

2vzi_B L281.B F365.B N273.B T270.B T252.B L333.B T366.B N363.B R359.B V294.B A257.B F250.B K355.B L299.B K265.B L315.B E304.B L303.B V308.B V283.B L361.B L253.B Y362.B H275.B L268.B F290.B V264.B L298.B G301.B N280.B V272.B V263.B L364.B F254.B L261.B F313.B L279.B D248.B F271.B T357.B I269.B T316.B L286.B Y306.B L302.B G305.B K260.B L358.B A249.B T267.B P309.B L310.B V297.B F307.B M300.B L296.B K278.B

2xl2_A V132.A Y252.A F222.A F137.A C135.A D92.A I262.A T269.A S267.A T312.A V48.A W273.A S189.A V275.A I229.A I305.A V93.A E322.A K259.A P224.A D192.A Y260.A D107.A L321.A A308.A S148.A A176.A F219.A N265.A F133.A V174.A S190.A L234.A K272.A N225.A S306.A Q289.A K52.A F149.A F266.A A47.A V217.A G271.A L230.A A65.A F53.A H178.A L240.A V220.A A264.A C309.A Y131.A K250.A N136.A S50.A W241.A V268.A T307.A P173.A S175.A S49.A Y228.A D172.A K221.A V187.A A94.A I274.A W95.A K227.A C134.A V51.A V177.A P311.A D242.A S54.A L288.A I90.A Y191.A F263.A H310.A S91.A L249.A G226.A P55.A C261.A S223.A

2xt1_A L190.A N183.A A209.A Q179.A C218.A L205.A I150.A L189.A Y169.A K170.A L172.A E187.A Y164.A Q155.A M185.A A174.A L211.A G208.A V165.A T210.A L202.A F168.A E212.A V191.A K199.A A194.A C198.A T186.A M214.A R173.A M215.A Q192.A F161.A R162.A G206.A K182.A D166.A P207.A

2ygg_B F142.B D57.B D21.B L113.B M146.B M73.B V143.B V56.B A16.B T29.B M125.B A74.B I126.B A104.B I28.B M72.B I64.B V36.B S18.B R107.B V109.B E105.B E128.B I86.B L117.B L19.B D130.B S102.B M110.B F90.B M145.B A129.B F20.B E15.B L33.B A103.B T80.B V137.B I101.B A89.B G135.B L106.B I53.B E12.B F17.B K78.B F13.B F93.B F69.B Q136.B M37.B V122.B N54.B L49.B D94.B M77.B E55.B R75.B L40.B K76.B K95.B H108.B M52.B R127.B

2zl1_B S21.B T40.B A95.B S50.B Y94.B I48.B V81.B W53.B L73.B T45.B D41.B G79.B L23.B A42.B M74.B M93.B A49.B T22.B D39.B D75.B A82.B C92.B Q20.B R80.B I83.B I52.B A46.B E78.B V44.B C76.B I72.B

2zn9_A H131.A Y183.A Q159.A G123.A F138.A S189.A I164.A Q172.A V157.A Y168.A F122.A R166.A Y124.A D132.A K137.A C155.A R160.A D173.A F165.A R125.A D169.A T162.A F130.A Q153.A L158.A V178.A G154.A D163.A W175.A V187.A Q182.A I133.A L115.A L161.A F188.A L134.A I135.A L119.A G174.A L126.A M186.A I176.A

2zpy_A I248.A L223.A S249.A H288.A I257.A L218.A W242.A N247.A F240.A L281.A G224.A M285.A V220.A L225.A E289.A I245.A F250.A R246.A M292.A P241.A I260.A I280.A S243.A F255.A A282.A N277.A C284.A G286.A

3aa6_B N24.B D149.B T186.B P18.B D139.B F112.B W148.B P17.B R244.B I239.B D44.B K142.B T172.B K237.B N188.B Y232.B M174.B R108.B G140.B I21.B F233.B Q19.B H152.B W176.B C147.B L193.B S42.B Y111.B L189.B L16.B G185.B S141.B L47.B I134.B I231.B V43.B S192.B D109.B Q45.B P46.B V240.B E221.B M13.B S226.B L10.B N222.B M187.B G138.B T236.B G191.B S150.B Q20.B I144.B A137.B G242.B R225.B G146.B Q178.B G183.B T179.B D238.B K181.B E113.B S184.B V173.B I151.B R15.B K235.B R14.B A9.B K136.B K145.B L228.B T194.B K135.B D11.B Y107.B N241.B L243.B L177.B G190.B N229.B E218.B V217.B L175.B

3aaj_B I133.B I174.B L132.B I188.B Y166.B F136.B V176.B G152.B L119.B Y122.B L159.B V155.B L156.B I131.B R123.B S125.B Y181.B V185.B T160.B R164.B F163.B I162.B H129.B M184.B L124.B F128.B K135.B Q127.B R158.B

3av1_D R69.D L99.D R89.D L98.D V108.D L103.D T87.D T93.D I66.D R96.D S109.D T116.D T112.D I86.D S88.D K113.D E68.D I91.D F67.D V95.D A114.D G111.D Q92.D A104.D A107.D E110.D F62.D A71.D I70.D T119.D V115.D

3av1_E Q93.E R63.E L103.E A98.E T58.E Q55.E D106.E S57.E F67.E I51.E S96.E R52.E I62.E I119.E T107.E E97.E R53.E I112.E V117.E E105.E T118.E G102.E L61.E N108.E L100.E V101.E F104.E L70.E E59.E I124.E L60.E Y54.E E50.E

3av1_H H106.H S88.H E110.H I86.H A71.H V63.H A104.H T112.H S109.H I66.H F62.H L103.H V95.H A74.H V108.H Y34.H N64.H I91.H F67.H A107.H G111.H L99.H I70.H E68.H Q92.H

3azg_D A74.D R69.D L99.D R89.D L98.D V108.D L103.D V63.D T87.D I66.D Y118.D S109.D T116.D T112.D S88.D I86.D K113.D E68.D I91.D F67.D V95.D A114.D G111.D Q92.D A104.D A107.D E110.D H106.D F62.D A71.D I70.D T119.D V115.D

3azg_H H106.H G50.H S88.H V41.H S35.H E110.H V115.H K40.H T49.H I86.H V63.H A71.H S52.H T112.H S109.H K43.H Y39.H T87.H M56.H I66.H V45.H I36.H F62.H L103.H L42.H V95.H H46.H A74.H Y34.H V108.H N64.H A55.H I58.H I91.H F67.H A114.H N60.H A107.H G111.H L99.H I70.H I51.H R89.H E32.H E68.H V38.H Y37.H M59.H Q92.H S33.H

3b3g_A M164.A H275.A G241.A E215.A Q159.A M269.A V192.A C194.A L190.A L157.A G195.A L274.A S196.A L226.A A223.A Q161.A Q221.A E224.A Q205.A A220.A L199.A K228.A H222.A S257.A S200.A A213.A L232.A Y156.A G197.A I255.A G193.A I211.A V238.A I236.A Q447.A E258.A I239.A M219.A V227.A K242.A P259.A N230.A S229.A F201.A M163.A L270.A S217.A D191.A S272.A A204.A Q160.A Y273.A V214.A E245.A G155.A V243.A I198.A R268.A V237.A N231.A A216.A K471.A E244.A A276.A E267.A N266.A M260.A

3bya_A L112.A L116.A E114.A V91.A L18.A F68.A E11.A M144.A M36.A Q41.A L39.A R126.A D129.A F12.A E14.A V121.A A88.A F141.A I125.A F89.A V35.A E127.A A128.A I100.A V108.A M124.A S38.A Q135.A M109.A T146.A N111.A V136.A S101.A T110.A G40.A A15.A L105.A R106.A E87.A M145.A F16.A F92.A F19.A

3fdl_A L112.A F144.A K87.A D189.A E179.A R139.A V141.A G138.A L108.A A149.A L90.A W181.A R103.A E129.A V86.A S145.A M83.A V126.A L174.A I166.A F97.A I140.A Q111.A A171.A R91.A M170.A L150.A T109.A G94.A F123.A F105.A L194.A F191.A F143.A N136.A A142.A A168.A E98.A T172.A Q125.A G187.A F146.A L130.A N175.A Q88.A S122.A W188.A H113.A Y101.A L99.A S110.A V127.A I114.A S106.A R132.A L178.A R102.A A104.A G147.A A167.A W137.A I182.A

3fdm_B I166.B F191.B Y173.B Q26.B G187.B T172.B I140.B A171.B L174.B Q88.B M170.B A168.B F144.B L178.B N175.B F131.B K87.B M83.B V192.B W188.B A167.B V141.B W181.B V135.B I182.B W137.B D189.B R91.B L90.B Q183.B E179.B F143.B V86.B

3gmo_A T156.A L116.A F171.A Q99.A Y73.A L66.A L170.A T37.A R74.A I147.A I98.A P169.A W133.A M162.A Q161.A L143.A V118.A G103.A V172.A A102.A G56.A V126.A R127.A R79.A S59.A K57.A Q55.A V125.A S28.A Q67.A A119.A L150.A L175.A I81.A C168.A D80.A Q62.A L58.A T167.A T159.A D153.A L164.A L13.A N60.A S101.A L163.A M69.A F70.A Q14.A L100.A E64.A R173.A V160.A G155.A V72.A S157.A V30.A S54.A W40.A F115.A G174.A F77.A S76.A H38.A D166.A I47.A H117.A W63.A C12.A F10.A

3gst_A S138.A V155.A F183.A L180.A E132.A L136.A V99.A Y137.A I98.A Q102.A V103.A K133.A D161.A N73.A I111.A Q71.A V9.A M134.A I94.A P60.A I162.A M104.A L211.A D105.A G11.A Y160.A L59.A M112.A N58.A M108.A F147.A L12.A L110.A Y115.A M34.A I207.A N116.A Y6.A L158.A L163.A L141.A R107.A R95.A E100.A Q109.A Q165.A S72.A W7.A A96.A S209.A F208.A R10.A N106.A R42.A A159.A F140.A

3h53_A V395.A L261.A W222.A M270.A S227.A L226.A W273.A R344.A F341.A E325.A R316.A L272.A V233.A S393.A L323.A S266.A V225.A A268.A E264.A W223.A N230.A Y327.A K319.A D346.A C343.A G394.A T345.A Q265.A E234.A Q269.A S342.A P392.A L229.A

3hr0_A Q619.A L548.A L675.A S536.A L611.A F544.A A540.A N612.A L545.A T537.A V546.A V551.A L608.A D538.A M542.A I616.A T614.A L604.A G607.A A615.A L603.A E606.A F663.A T547.A P618.A E610.A K541.A L667.A

3ikp_B V231.B E232.B K246.B K229.B Y228.B G266.B C353.B S239.B A264.B F225.B C261.B I244.B E354.B F245.B K230.B G241.B S226.B F355.B G265.B K243.B A224.B Q227.B Q222.B L233.B E242.B

3io9_A A272.A T266.A F270.A I237.A R263.A ZN3.A S247.A L246.A S293.A I268.A H252.A F254.A I264.A V274.A L186.A H224.A L232.A L267.A G271.A V220.A V297.A L290.A M250.A G230.A V253.A G262.A A227.A S245.A I251.A I294.A V243.A F228.A V249.A N260.A M231.A K234.A L235.A A275.A

3kj0_A A272.A T266.A F270.A I237.A R263.A S247.A L246.A K244.A S293.A I268.A H252.A F254.A I264.A V274.A L186.A H224.A L232.A L267.A G271.A K302.A V297.A L290.A M250.A L298.A G230.A N239.A S269.A V253.A G262.A A227.A I251.A I294.A V243.A F228.A V249.A D242.A T259.A N260.A M231.A L278.A L235.A K234.A

3kz0_A A272.A T266.A F270.A I237.A R263.A S247.A L246.A K244.A S293.A I268.A H252.A V274.A L186.A L232.A A291.A L267.A G271.A L290.A M250.A P289.A G230.A S269.A V253.A I251.A I294.A V243.A F228.A V249.A M231.A L278.A L235.A K234.A A275.A

3lk4_B D149.B T186.B D139.B Y64.B F112.B W148.B K48.B D44.B K142.B T172.B K237.B L25.B V153.B N188.B D7.B Y232.B M174.B G140.B G234.B F233.B H152.B W176.B C147.B L61.B L193.B S42.B Y111.B L189.B T227.B S141.B L47.B I134.B S41.B T170.B V43.B R66.B C8.B S192.B Q45.B P46.B E230.B V240.B E221.B M13.B S226.B L10.B N222.B M187.B G138.B T236.B S150.B L133.B I144.B A137.B R225.B G146.B S171.B Q178.B I224.B V131.B T179.B K181.B E113.B V173.B D63.B I151.B L60.B Y71.B R14.B K136.B C62.B L228.B K145.B T194.B K143.B L40.B E22.B K135.B I29.B D11.B L177.B N229.B I132.B L39.B L6.B L175.B

3maz_A Y227.A M230.A Y211.A E259.A Q217.A I201.A E241.A N199.A I215.A R265.A K228.A T237.A E218.A V257.A K225.A T214.A I253.A I238.A Y255.A S212.A F256.A T260.A L240.A K242.A L202.A E239.A V229.A R216.A R261.A V252.A N263.A M200.A I213.A I219.A L264.A

3mk8_A A272.A T266.A F270.A I237.A R263.A S247.A L246.A S293.A I268.A H252.A F254.A I264.A V274.A L186.A L232.A A291.A L267.A G271.A L290.A M250.A P289.A V253.A I294.A V243.A F228.A V249.A M231.A L278.A L235.A A275.A

3nqu_A K64.A E99.A A100.A F101.A V103.A L61.A F106.A V126.A A71.A Y110.A L94.A F67.A E96.A L121.A R72.A H59.A E107.A R63.A L70.A Q95.A I62.A L102.A L105.A H104.A L60.A I74.A A98.A

3o2q_A A235.A L228.A M164.A I203.A R206.A L170.A Y137.A V192.A I191.A L190.A F242.A M133.A T193.A N262.A E230.A T134.A K241.A L172.A K233.A P204.A R265.A L131.A D167.A G232.A R205.A D210.A H245.A E231.A I211.A S212.A L236.A A183.A D173.A L240.A V161.A Q135.A L194.A V227.A I130.A E188.A G166.A L239.A Q266.A I169.A L141.A V187.A W229.A I184.A E237.A K185.A K138.A I168.A R267.A S162.A Q238.A W158.A A163.A H209.A Q207.A A165.A R197.A A234.A F186.A G189.A H182.A

3o76_A P53.A I203.A V10.A T131.A F129.A N93.A I107.A H125.A P128.A V92.A N206.A V104.A M39.A I161.A S65.A D98.A E130.A R13.A I143.A R100.A V144.A F8.A D94.A K44.A G12.A Q135.A W38.A Y108.A N204.A L158.A L132.A L176.A P202.A M89.A I35.A K102.A L52.A T34.A G124.A Q51.A L155.A Q88.A Y103.A Y49.A L99.A D90.A R11.A A87.A M91.A G50.A P9.A V33.A G95.A Y7.A D36.A V96.A L159.A L126.A Q64.A G205.A

3onz_B Y130.B L141.B N139.B L81.B V67.B L110.B L105.B T87.B A140.B F42.B HEM148.B D99.B K66.B A86.B D73.B V1.B L28.B V134.B A138.B F85.B F103.B T84.B S72.B V137.B R104.B H63.B F122.B L114.B F41.B L106.B A70.B N108.B N102.B L78.B T38.B C112.B V98.B Q131.B G74.B L75.B A27.B L68.B F71.B Q127.B V111.B V11.B A135.B V133.B L31.B G107.B

3qrd_B V23.B K54.B A16.B R45.B G22.B V49.B V31.B A52.B F29.B A30.B E21.B R51.B L47.B L27.B V18.B S17.B E19.B V50.B Y34.B Q48.B R24.B A46.B D28.B A26.B R53.B R25.B E20.B

3r85_A L112.A W169.A F144.A V141.A R139.A G138.A L108.A A149.A R103.A E129.A S145.A V126.A V135.A L174.A E153.A I166.A G134.A F97.A I140.A Q111.A A171.A Y173.A M170.A L150.A T109.A G94.A F123.A F105.A F143.A E124.A A142.A E98.A N128.A Q125.A C151.A F146.A L130.A S122.A Y101.A L99.A V127.A I114.A S106.A L178.A R102.A A104.A G147.A F131.A A167.A

3r8i_A K301.A L228.A F363.A Y473.A I472.A L340.A K457.A C285.A L469.A E295.A I456.A L255.A P227.A L353.A I262.A E259.A Q294.A H449.A R288.A Q283.A G321.A T229.A E343.A I279.A V322.A L452.A L468.A H466.A K367.A Q286.A V339.A V307.A G284.A I296.A A292.A Q470.A F282.A V290.A S464.A E471.A V277.A V293.A M364.A T461.A I325.A T459.A I249.A F368.A T297.A T316.A H266.A M329.A L476.A E291.A P467.A V455.A R280.A Q314.A L317.A Y320.A I392.A I326.A S289.A G258.A M348.A K319.A Y327.A F306.A N312.A F352.A F264.A H323.A E460.A I341.A V315.A V446.A F360.A Q454.A D475.A M334.A K263.A F226.A L453.A A300.A K474.A L333.A K265.A F287.A L318.A L330.A G344.A I281.A S342.A K458.A D462.A E298.A L311.A L465.A M463.A

3rnm_A G214.A K87.A A365.A V364.A I358.A M65.A V188.A Y62.A Y63.A V357.A V48.A G215.A M326.A C45.A S356.A D86.A F71.A I189.A S168.A I76.A E77.A N84.A E80.A G217.A Y359.A S60.A Q91.A H61.A G49.A G279.A N58.A M78.A S53.A N59.A S79.A P325.A A328.A R280.A R82.A S150.A L85.A T44.A I218.A L327.A L200.A K54.A D320.A H329.A P16.A V81.A W366.A A66.A E192.A V152.A G324.A V216.A L57.A C50.A M88.A L83.A H67.A

3u2v_A A235.A D125.A T11.A N133.A Y187.A K76.A A122.A V63.A S196.A A123.A G215.A L189.A D104.A P184.A T8.A L80.A I194.A P126.A T83.A V82.A K218.A F185.A L167.A R77.A E79.A A206.A S190.A H212.A N188.A W220.A S193.A V217.A G81.A F204.A T10.A L9.A T85.A F100.A H236.A A208.A P129.A W174.A P78.A Y15.A Y197.A P238.A L183.A L198.A W128.A K86.A V203.A M75.A N12.A D102.A R216.A S134.A F201.A V136.A N239.A I192.A T223.A K209.A D163.A N221.A L214.A A200.A G127.A M240.A Y195.A A103.A V210.A Y222.A G135.A I186.A P199.A Q42.A F245.A F213.A S207.A M101.A E234.A D160.A D237.A Q164.A S191.A H182.A P124.A G205.A S233.A K232.A

3uyj_B T244.B N359.B H352.B D323.B P324.B W414.B Q322.B H321.B Y272.B A309.B S241.B V402.B L273.B V239.B K397.B N308.B S318.B L329.B W248.B I394.B W310.B Q275.B V270.B D245.B P319.B R242.B A274.B I317.B K336.B I338.B T316.B S412.B G240.B H400.B L320.B N327.B Y243.B S410.B G271.B E238.B H276.B T360.B

3zwh_B A54.B Y19.B K48.B L29.B F72.B L37.B E41.B V70.B S86.B T39.B L62.B L38.B L42.B F16.B D51.B V77.B K35.B S80.B Y75.B R49.B T15.B K7.B G47.B E36.B P43.B M85.B K57.B A83.B R40.B F78.B S44.B F55.B L9.B V13.B T50.B L34.B E52.B E74.B S81.B I82.B C76.B M12.B V11.B A8.B L58.B W45.B L79.B L46.B

4dmb_B A54.B L113.B S47.B I109.B Y51.B M50.B R72.B L121.B D48.B A74.B G25.B T110.B Q26.B V46.B S45.B L17.B L73.B K28.B L117.B L21.B L27.B F20.B E120.B V71.B L124.B D78.B L18.B M79.B D116.B V24.B L75.B V76.B R52.B A80.B R118.B

4dra_H F45.H K12.H A50.H V15.H V47.H L19.H V44.H E40.H F10.H K43.H S8.H L42.H L14.H L18.H V46.H V39.H G9.H R11.H H22.H A6.H M38.H G7.H

4f3l_A E326.A G332.A E367.A E283.A I357.A L321.A Y355.A R365.A Y359.A L282.A L328.A S281.A F368.A A322.A Q330.A M329.A C371.A I369.A H280.A C324.A P366.A L287.A S308.A T358.A H325.A K285.A T353.A Y313.A Y310.A W284.A Y356.A L318.A D311.A G309.A

4gst_B G11.B V103.B A159.B M104.B L141.B E139.B W7.B F147.B A96.B L59.B M134.B Y115.B R107.B L163.B L110.B L136.B Q109.B F208.B V9.B S209.B K210.B N106.B V155.B D161.B Q165.B M34.B L158.B F140.B K133.B R95.B I207.B I111.B E132.B M108.B I162.B Q102.B S138.B Y137.B L180.B R10.B V99.B L211.B F183.B Y6.B Y160.B I98.B N8.B W45.B L12.B R42.B

5gst_A S138.A V155.A F183.A L180.A E132.A L136.A V99.A Y137.A I98.A Q102.A V103.A K133.A D161.A N73.A I111.A Q71.A V9.A M134.A K210.A I94.A P60.A I162.A M104.A L211.A D105.A G11.A Y160.A L59.A W45.A N58.A M108.A F147.A L12.A L110.A Y115.A M34.A Y166.A I207.A W214.A Y6.A L158.A L163.A L141.A R107.A R95.A E139.A Q109.A Q165.A S72.A W7.A A96.A S209.A F208.A R10.A N106.A R42.A A159.A F140.A C114.A

5gst_B V103.B A159.B M104.B L141.B E139.B F147.B A96.B Y166.B M134.B L163.B L136.B Q109.B N106.B V155.B D161.B L158.B F140.B K133.B D156.B R95.B E100.B E132.B I162.B Q102.B Y137.B L180.B V99.B I94.B F183.B Y160.B I98.B K135.B A148.B D105.B

6gst_A S138.A V155.A L180.A E132.A L136.A V99.A Y137.A I98.A Q102.A V103.A K133.A M134.A I94.A I162.A Y160.A F147.A Y166.A I130.A L158.A L163.A L141.A R107.A R95.A E139.A Q109.A A96.A N106.A A159.A F140.A

6gsu_A S138.A V155.A F183.A L180.A E132.A L136.A V99.A Y137.A I98.A Q102.A V103.A K133.A D161.A N73.A I111.A Q71.A V9.A A13.A M134.A K210.A I94.A P60.A I162.A M104.A K49.A L211.A D105.A G11.A Y160.A L59.A W45.A A148.A N58.A N8.A M108.A F147.A L12.A L110.A Y115.A M34.A I207.A Y6.A L158.A K135.A L163.A L141.A R107.A R95.A E100.A E139.A Q109.A Q165.A S72.A W7.A A96.A S209.A F208.A R10.A N106.A R42.A A159.A F140.A

6gsv_B G11.B M112.B V103.B A159.B M104.B L141.B E139.B W7.B F147.B A96.B L59.B Y166.B M134.B Y115.B R107.B L163.B L110.B Y40.B L136.B F208.B Q109.B V9.B S209.B K210.B N73.B N106.B V155.B D161.B Q165.B M34.B L158.B Q213.B F140.B K133.B R95.B A212.B S72.B I207.B I111.B E132.B M108.B I162.B Q102.B S138.B Y137.B L180.B R10.B V99.B L211.B I94.B F183.B Y6.B Y160.B P60.B I98.B N8.B A148.B L12.B R42.B N58.B D105.B

6gsx_B P206.B F6.B G11.B A33.B V103.B A159.B M104.B L141.B E139.B W7.B Y32.B F147.B A96.B L59.B M134.B Y115.B R107.B L163.B L110.B L136.B Q109.B F208.B V9.B S209.B N73.B K210.B N106.B V155.B Q165.B M34.B L158.B F140.B K133.B R95.B D36.B S72.B I207.B I111.B E132.B M108.B I162.B Q102.B S138.B Y137.B L180.B R17.B R10.B V99.B L211.B I94.B T13.B Y160.B P60.B I98.B N8.B A148.B W45.B L12.B R42.B N58.B D105.B
