# Supplementary material for: Structure-Based Druggability Assessment of the Mammalian Structural Proteome with Inclusion of Light Protein Flexibility
Source: PLoS Comput Biol. 2014 Jul 31;10(7):e1003741. doi: 10.1371/journal.pcbi.1003741 (PMC4117425; doi:10.1371/journal.pcbi.1003741)
Supplement: Table S4 — Extension of Table 2 to comparison of druggability estimations on all targets from Huang and Jacobson [17] . The data under the Structural data and Docking-based druggability are from reference 17. Aldose reductase sites required manual intervention to include NAP co-factor. Without co-factor, Dscore+ is lower, around 1.1. The DHFR structure with PDB ID 6dfr is missing a large portion of the binding site (both protein and co-factor), and so calculations would not be relevant; we indicated this with “[Not calculated]”. The flexible druggability method is only performed for binding sites that meet an initial score (with the rigid crystal structure). However, for the purposes of this study, we removed this cut-off in order to generate values for IL-2 and HPV E2. For IL-2, performing the flexibility modeling procedure results in DScore+ values of 1.5 (1z92), 1.5 (1py2), and 1.7 (1m48), with small, non-drug-like volumes of 98, 82, 53, respectively. For HPV E2, the DScore+ values are 1.1 (1tue) and 1.5 (1r6n), with reasonable drug-like volumes. For neuraminidase (NA), the Dscore+ values are 1.8 (1a4g), 1.7 (1a4q), and 1.7 (1nsc), with drug-like volumes. (DOCX) [file pcbi.1003741.s004.docx]

| **Target** | **Structural data** | | **Docking-based druggability** | **Rigid crystal** | **Protein flexibility** |
| --- | --- | --- | --- | --- | --- |
|  | PDB ID | RMSD_ave_ (Å) | docking hit rate | DScore+ | DScore+ w/ IFD |
| ACE | 1uze |  | 0.60 | 1.3 | 1.6 |
| (difficult) | 1o66 | 0.2 | 0.45 | 1.3 | 1.3 |
|  | 1uzf | 0.4 | 0.69 | 1.3 | 1.6 |
| ALR2 | 1ah0 |  | 1.42 | 1.4 | 1.9 |
|  | 1ah3 | 1.1 | 1.27 | 1.4 | 1.8 |
|  | 2acr (apo) | 0.9 | 1.10 | 1.4 | 1.6 |
| CDK2 | 1aq1 |  | 1.32 | 1.3 | 1.7 |
|  | 1buh (apo) | 1.8 | 1.44 | 1.5 | 1.7 |
|  | 1dm2 | 1.8 | 1.62 | 1.3 | 1.9 |
| COX-2 | 1cvu |  | 1.51 | 1.7 | 2.4 |
|  | 1cx2 | 1.2 | 1.53 | 1.9 | 2.0 |
|  | 3pgh | 1.1 | 1.64 | 2.0 | 2.2 |
| DHFR | 3dfr |  | 1.01 | 1.5 | 1.8 |
|  | 6dfr (apo) | 1.5 | 1.02 | [See caption] | [See caption] |
| ER | 1l2i |  | 1.69 | 2.4 | 2.9 |
|  | 3ert | 2.6 | 1.55 | 2.0 | 2.7 |
|  | 1err | 2.0 | 1.61 | 2.1 | 2.8 |
| Fxa | 1f0r |  | 1.64 | 1.3 | 1.7 |
|  | 1fjs | 1.1 | 1.59 | 1.3 | 1.7 |
|  | 1ksn | 0.7 | 1.59 | 1.3 | 1.8 |
|  | 1xka | 1.3 | 1.56 | 1.3 | 1.7 |
| HIVRT | 1vrt |  | 1.66 | 2.5 | 2.5 |
|  | 1rt1 | 1.5 | 1.75 | 1.6 | 2.3 |
|  | 1c1c | 1.9 | 1.61 | 1.6 | 2.2 |
|  | 1rth | 1.6 | 1.61 | 1.7 | 2.3 |
| HMGR | 1hw8 |  | 1.39 | 1.4 | 1.7 |
|  | 1hwk | 0.6 | 1.31 | 1.4 | 1.9 |
| NA | 1a4g |  | 0.57 | 1.2 | * |
|  | 1a4q | 0.5 | 0.52 | 1.2 | * |
|  | 1nsc | 0.3 | 0.52 | 1.2 | * |
| P38a | 1a9u |  | 1.00 | 1.4 | 1.8 |
|  | 1kv1 | 3.8 | 1.16 | 1.6 | 2.1 |
|  | 1kv2 | 3.5 | 1.61 | 1.8 | 2.1 |
| PDE5 | 1xoz |  | 1.18 | 1.5 | 1.8 |
|  | 1xp0 | 0.8 | 1.24 | 1.4 | 1.8 |
| PPARg | 1fm6 |  | 1.46 | 1.8 | 2.9 |
|  | 1fm9 | 1.5 | 1.62 | 2.6 | 3.0 |
|  | 2prg | 0.7 | 1.43 | 1.9 | 2.1 |
| Thrombin | 1ba8 |  | 1.53 | 1.3 | 1.8 |
|  | 1hgt (apo) | 0.7 | 1.55 | 1.3 | 1.8 |
| TK | 1kim |  | 1.58 | 1.7 | 2.7 |
|  | 1ki4 | 1.8 | 1.40 | 1.8 | 2.6 |
| IL-2 | 1z92 |  | 0.13 | 0.9 | * |
|  | 1py2 | 2.6 | 0.62 | 1.2 | * |
|  | 1m48 | 2.5 | 0.62 | 1.1 | * |
| Bcl-XL | 2bzw |  | 1.04 | 1.5 | 2.4 |
|  | 2yxj | 2.5 | 0.84 | 1.8 | 2.5 |
| TNF | 1tnf |  | 0.95 | 1.8 | 2.4 |
|  | 2az5 | 2.9 | 0.96 | 1.7 | 2.0 |
| MDM2 | 1ycr |  | 0.45 | 1.7 | 2.5 |
|  | 1rv1 | 1.8 | 0.92 | 1.7 | 2.2 |
|  | 1t4e | 1.6 | 0.66 | 1.6 | 2.1 |
| HPV E2 | 1tue |  | -0.24 | 1.0 | * |
|  | 1r6n | 2.8 | 1.02 | 1.2 | * |
| ZipA | 1f47 |  | -0.02 | 0.9 | * |
|  | 1y2f | 0.6 | -0.10 | 0.9 | * |

**Table S4. Extension of Table 2 to comparison of druggability estimations on all targets from *Huang and Jacobson*** [17]. The data under the *Structural data* and *Docking-based druggability* are from reference 17. Aldose reductase sites required manual intervention to include NAP co-factor. Without co-factor, Dscore+ is lower, around 1.1. The DHFR structure with PDB ID 6dfr is missing a large portion of the binding site (both protein and co-factor), and so calculations woul not be relevant; we indicated this with “[Not calculated]”. The flexible druggability method is only performed for binding sites that meet an initial score (with the rigid crystal structure). However, for the purposes of this study, we removed this cut-off in order to generate values for IL-2 and HPV E2. For IL-2, performing the flexibility modeling procedure results in DScore+ values of 1.5 (1z92), 1.5 (1py2), and 1.7 (1m48), with small, non-drug-like volumes of 98, 82, 53, respectively. For HPV E2, the DScore+ values are 1.1 (1tue) and 1.5 (1r6n), with reasonable drug-like volumes. For neuraminidase (NA), the Dscore+ values are 1.8 (1a4g), 1.7 (1a4q), and 1.7 (1nsc), with drug-like volumes.
